# MultiCaRe-Thorax: A Curated Benchmark Dataset and Reproducible Labeling Framework for Multi-Label Thoracic Disease Classification in Chest X-Ray Imaging

**DOI:** 10.3390/jimaging12100484

**Published:** 2026-10-05

**Authors:** Nour J. Abu Jassar, Islam T. Almalkawi, Mohammad F. Al-Hammouri, Tassniem Omari, Ali Al Bataineh

**Affiliations:** 1 Department of Computer Engineering, Faculty of Engineering, The Hashemite University, Zarqa 13133, Jordan; eslam.malkawi@hu.edu.jo (I.T.A.); alhammouri@hu.edu.jo (M.F.A.-H.); 2 Department of Electrical Engineering, Faculty of Engineering, The Hashemite University, Zarqa 13133, Jordan; tassniem@hu.edu.jo; 3 Computer Information Science, Higher Colleges of Technology, Abu Dhabi 25026, United Arab Emirates; aalbataineh@hct.ac.ae

**Keywords:** chest X-ray, thoracic diseases, multi-label classification, deep learning, convolutional neural networks, medical imaging, artificial intelligence

## Abstract

Chest X-ray (CXR) imaging remains a widely used diagnostic modality for the detection of thoracic diseases, including pneumonia, tuberculosis, pleural effusion, lung cancer and cardiomegaly. Despite the extensive use of CXR examinations in clinical practice, the development of robust multi-label artificial intelligence (AI) systems remains constrained by limitations in dataset availability, annotation consistency and reproducible labeling methodologies. Existing public datasets frequently rely on heterogeneous labeling protocols, limited pathology coverage or annotations that inadequately account for linguistic negation and uncertainty within radiology reports. In this paper, we introduce MultiCaRe-Thorax, a curated thoracic benchmark dataset derived from the publicly available MultiCaRe repository, with a reproducible negation-aware natural language processing (NLP) framework for automated generation of multi-label thoracic disease annotations. Unlike existing CXR benchmarks that are distributed in their pre-curated form, MultiCaRe-Thorax provides a fully reproducible pipeline from raw case reports to curated dataset, establishing a new resource that is both scalable and transparent. Our pipeline performs thoracic case identification, image–report matching, duplicate removal, data cleaning, disease-specific label extraction and quality-control verification to produce a curated cohort comprising 5252 chest radiographs from 3415 unique patients. Sixteen clinically relevant thoracic pathologies were automatically annotated using a dictionary- and pattern-based NLP framework incorporating explicit negation detection and n-gram mining, achieving 95% accuracy against 500 manually reviewed reports (Cohen’s κ=0.89). Sixteen clinically relevant thoracic pathologies were automatically annotated using a dictionary- and pattern-based NLP framework incorporating explicit negation detection and n-gram mining, achieving 95% agreement against 500 manually reviewed reports. Five ImageNet-pretrained convolutional neural network (CNN) architectures (ResNet50, DenseNet121, EfficientNetB0, EfficientNetB7 and InceptionV3) with two hybrid ensemble configurations were evaluated under a patient-level data partitioning protocol. Among individual architectures, DenseNet121 attains the best AUC on tuberculosis (0.757) and pleural effusion (0.767) with roughly one-third the parameters of ResNet50, while ResNet50 attains the higher mean AUC across all sixteen pathologies (0.688 vs. 0.682) and on interstitial lung disease (0.769) and lung mass/cancer (0.777); we report this as an accuracy–efficiency trade-off rather than a single best architecture. The DenseNet121-InceptionV3 ensemble achieves the highest reported AUC of 0.821 for lung mass/cancer detection. We report 95% confidence intervals for the evaluated ensemble and remaining backbones and note substantial estimation uncertainty for the rarest classes (e.g., congenital heart disease, n=8 test cases). Grad-CAM visualizations were also employed to provide clinically interpretable explanations of model predictions.

## 1. Introduction

Chest X-ray (CXR) imaging represents the foundational diagnostic modality for detecting thoracic diseases, including pneumonia, tuberculosis, lung cancer, and cardiomegaly, owing to its wide availability, non-invasive nature, and cost-effectiveness [1,2,3,4,5,6,7,8,9]. The sheer volume of CXRs interpreted annually, estimated to reach hundreds of millions worldwide, underscores their pivotal role in public health and clinical decision-making. Despite modern advancements in imaging technology, CXR remains indispensable, particularly for early identification of respiratory diseases, mass screening programs, and resource-limited environments [10,11,12]. The diagnostic power of chest radiographs is constrained by intrinsic challenges. The two-dimensional projection of complex three-dimensional thoracic anatomy results in overlapping anatomical structures, low soft tissue contrast, and subtle pathological findings, rendering accurate visual interpretation non-trivial [7,13,14,15,16,17]. Moreover, multiple coexisting thoracic pathologies within a single radiograph introduce additional complexity, as manifestation patterns frequently overlap among diseases. Given these factors and reported intra- and inter-observer variability among radiologists [18,19], the demand for robust, automated, and scalable diagnostic tools has intensified.

Artificial Intelligence (AI), particularly deep learning and convolutional neural networks (CNNs), has rapidly transformed medical image interpretation [20,21,22,23]. These models learn discriminative features directly from pixel data, often matching or surpassing human expert performance in certain diagnostic tasks. Within the AI-driven clinical paradigm, focus has shifted toward multi-label classification frameworks, as opposed to traditional single-label or binary classifiers, to better reflect clinical realities where multiple disease processes commonly coexist within a single radiograph [24].

Despite remarkable progress, current CXR diagnostic systems suffer from substantial limitations. The majority of existing AI models are optimized for single-label or binary classification, treating each thoracic disease as an independent task [1,10,25,26]. This oversimplification fails to account for frequent comorbidities and label dependencies, ultimately limiting diagnostic accuracy. Manual interpretation remains time-consuming, labor-intensive, and susceptible to human error and fatigue [27]. In high-volume settings, this increases report turnaround time and risks missed or delayed diagnoses of critical diseases [28,29]. Large-scale, expertly annotated multi-label CXR datasets are prohibitively expensive; consequently, automated labeling leveraging Natural Language Processing (NLP) techniques to extract disease labels from radiology reports has become standard practice [1,30,31,32]. However, many automated annotation pipelines inadequately address linguistic negation, contextual ambiguity, and uncertainty management, introducing label noise that can adversely affect downstream model development and evaluation [30,31,32].

In parallel with advances in AI methodologies, the research community has increasingly recognized the importance of high-quality benchmark datasets. Widely adopted resources such as ChestX-ray14, CheXpert, PadChest, and MIMIC-CXR have accelerated progress in thoracic image analysis by enabling standardized evaluation and comparison across studies [1,30,32]. Nevertheless, differences in annotation strategies, pathology definitions, labeling quality, and patient populations continue to complicate direct comparison among models and limit reproducibility across datasets. The recent release of the MultiCaRe dataset provides a new opportunity to investigate chest radiograph analysis within a contemporary multimodal clinical resource consisting of case reports and associated medical images spanning multiple anatomical regions and clinical specialties [33,34]. To date, however, no study has systematically derived, curated, labeled, and benchmarked a thorax-specific subset from the broader MultiCaRe repository. Three specific gaps motivate this work. First, no prior study has systematically derived, labeled, and benchmarked a thorax-specific subset of the MultiCaRe repository. Second, established negation-aware labelers (e.g., the CheXpert labeler [30], PadChest’s BiLSTM pipeline [32]) were developed for different report styles and are not directly portable to MultiCaRe’s case-report text. Third, this leaves no benchmarked link between a large, contemporary multimodal case-report resource and multi-label thoracic disease-classification results. We address these gaps by (1) curating MultiCaRe-Thorax, (2) developing a negation-aware NLP pipeline tailored to MultiCaRe’s report structure, and (3) establishing the first multi-architecture benchmark on this resource, including cross-dataset validation on ChestX-ray14. To address these limitations, this work introduces *MultiCaRe-Thorax*, a curated thoracic benchmark dataset derived from the publicly available MultiCaRe repository [33,34]. The proposed resource was constructed through a reproducible multi-stage pipeline involving thoracic case identification, image–report matching, duplicate detection, data cleaning, quality-control verification, and automated disease annotation. The resulting dataset comprises 5252 chest radiographs from 3415 unique patients and provides a dedicated benchmark resource for multi-label thoracic disease classification.

A second contribution of this work is the development of a reproducible negation-aware NLP framework for automated generation of disease labels from accompanying clinical case reports. The framework combines dictionary-based terminology matching, disease-specific pattern recognition, n-gram mining, and explicit negation detection to generate multi-label annotations for sixteen clinically relevant thoracic pathologies. By explicitly addressing linguistic negation and contextual ambiguity, the proposed approach improves annotation reliability while maintaining scalability for large collections of radiology reports.

Finally, the curated dataset is used to establish the first benchmark study on the MultiCaRe-Thorax resource through systematic evaluation of five ImageNet-pretrained CNN architectures (ResNet50, DenseNet121, EfficientNetB0, EfficientNetB7, and InceptionV3) together with hybrid ensemble configurations. Beyond establishing baseline performance metrics, the benchmark provides a reproducible foundation for future investigations involving advanced architectures, multimodal learning frameworks, and explainable AI methodologies for thoracic disease classification.

The novel contributions of this manuscript are summarized as follows:

1. We present the first curated thoracic benchmark dataset derived from the MultiCaRe repository through thoracic case filtering, image–report matching, duplicate analysis, quality-control verification and dataset cleaning that result in a cohort of 5252 chest radiographs from 3415 unique patients.
2. We develop a reproducible negation-aware NLP annotation pipeline incorporating dictionary matching, disease-specific pattern recognition, n-gram mining and explicit negation handling to generate labels for sixteen thoracic disease categories.
3. We establish the first benchmark study on the MultiCaRe-Thorax dataset through systematic evaluation of five ImageNet-pretrained CNN architectures and multiple hybrid ensemble configurations under a patient-level evaluation protocol.
4. We integrate Grad-CAM visualization to provide transparent and clinically interpretable explanations of model predictions to support future deployment in real-world diagnostic environments.
5. We release a reusable research resource comprising the curated dataset, preprocessing pipeline, labeling framework and benchmark results to facilitate future research in multi-label thoracic disease classification and trustworthy medical AI [35,36].

A distinguishing aspect of our work lies in the derivation of *MultiCaRe-Thorax*, a curated thoracic benchmark dataset extracted from the broader MultiCaRe repository [33,34]. Unlike existing benchmark datasets that are distributed in a pre-curated form, the construction of MultiCaRe-Thorax required a dedicated multi-stage workflow involving thoracic case identification, image–report alignment, duplicate detection, quality-control verification and automated disease annotation. To the best of our knowledge, this is the first study to systematically derive, annotate and benchmark a thorax-focused subset from MultiCaRe. The resulting dataset and labeling framework establish a reproducible resource for future research in multi-label thoracic disease classification.

The remainder of this paper is organized as follows. Section 2 details the MultiCaRe-Thorax dataset, labeling framework, and benchmarking methodology. Section 3 presents experimental results. Section 4 provides discussion and comparative analysis. Section 5 concludes the paper and outlines future research directions.

### 1.1. Dataset Annotation and Labeling Methodologies

Label generation for CXR datasets is resource-intensive and technically challenging. Three principal strategies dominate the field. Manual expert annotation involves radiologist or physician review of images and reports, yielding high-quality gold-standard label subsets, though typically at small scale [32,37]. Automated NLP-based annotation dominates public datasets, with NIH ChestX-ray14, CheXpert, and MIMIC-CXR employing rule-based and deep learning NLP to parse free-text radiology reports into multi-hot label vectors [1,30]. The CheXpert labeler toolkit combines regular expressions, negation detection, and uncertainty logic [30], while PadChest utilizes BiLSTM attention models for entity recognition and UMLS mapping [32]. Negation detection has been extensively studied in clinical NLP, with tools such as NegBio [38] and CheXbert [39] providing sophisticated negation and uncertainty handling. The NegEx algorithm [40] established the foundation for negation detection in clinical texts. Hybrid and three-tier validation procedures employ multi-step approaches: initial annotation followed by peer review and head radiologist adjudication to ensure clinical validity [30]. Recent work has explored single positive label learning [41,42] and zero-shot approaches [43,44] to address annotation challenges.

### 1.2. CNN Architectures and Evaluation Metrics

DenseNet-121 emerged as the de facto backbone standard due to efficient feature reuse, multi-label support, and lower parameter counts [45,46]. EfficientNet variants [47] have demonstrated parameter-efficient multi-label learning with competitive AUC scores [48,49]. More recent architectures such as ConvNeXtV2 [49] has shown promise for multi-label CXR tasks. The community primarily adopts three complementary metrics: AUC-ROC computed per class and averaged, though it may mask poor performance on rare classes [26,50]. Accuracy at a fixed threshold and Hamming loss are also commonly reported [26].

### 1.3. State-of-the-Art Approaches and Contributions

Table 1, Table 2 and Table 3 provide comprehensive comparisons of recent multi-label CXR studies across datasets, architectures, key contributions, and performance metrics.

Kufel et al. [45] presented a transfer learning framework using EfficientNet-B1 fine-tuning on NIH ChestX-ray14, achieving mean AUC of 84.28% through custom train-validation-test splitting and binary cross-entropy loss. Hanif et al. [51] fine-tuned DenseNet121 using temperature-scaled focal ZLPR ranking loss with test-time augmentations, achieving 80.96% average AUC. Yang and Wu [52] introduced a hierarchical attention CNN achieving weighted F1 of 0.97 and AUC exceeding 0.94 on ChestX-ray14. Ucan et al. [48] benchmarked EfficientNet variants achieving 83.09% mean AUC. Zhang et al. [53] (CorLabelNet) achieved state-of-the-art accuracy (84.5% on ChestX-ray14, 85.3% on CheXpert).

### 1.4. Vision Transformers in Medical Imaging

The Vision Transformer (ViT) [77] offers a contrasting paradigm to CNNs: images are partitioned into patches, embedded, and processed via multi-head self-attention, enabling global interactions from the first layer. Touvron et al. [78] introduced DeiT, using CNN distillation for data efficiency. Liu et al. [79] proposed the Swin Transformer with shifted windows, which has been widely adopted in medical image segmentation [80] and classification [81]. Chen et al. [82] showed ViT encoders capture rich CT/MRI features via TransUNet. Steiner et al. [83] confirmed large-scale pre-training is critical for small-data transfer.

Park et al. [84] compared ViT-B/16, ViT-L/16, and DeiT against DenseNet-121 on ChestX-ray14, finding that ImageNet-21k initialization narrows the gap under matched protocols. However, they noted that ViT variants still underperformed DenseNet-121 on several pathologies when evaluated under patient-level splits. Chen et al. [85] recently showed that ensemble approaches combining DenseNet and Swin Transformer can improve chest X-ray classification performance. Kim [86] proposed effective fusion of multi-view features using transformers for long-tailed chest X-ray classification. Naseer et al. [87] identified intriguing properties of vision transformers, including their ability to capture global context and their sensitivity to patch size and positional encoding. Tuli et al. [88] compared CNNs and transformers from a human vision perspective, finding that transformers exhibit more global processing while CNNs rely on local texture features.

These advances highlight the complementary strengths of CNNs and transformers, motivating future benchmark extensions on datasets such as MultiCaRe-Thorax.

### 1.5. Foundation Models and Self-Supervised Learning

Recent advances in self-supervised and foundation models have demonstrated promise for medical imaging. CheXzero [44] achieved expert-level detection from unannotated CXR images using contrastive learning with paired reports, performing on par with human radiologists on an external validation set [44]. Self-supervised pretraining strategies for CXR have shown improved performance on downstream tasks with limited labeled data. For example, CheSS (Chest X-Ray Pre-trained Model via Self-supervised Contrastive Learning) demonstrated a 28.5% increase in accuracy on a 6-class classification task compared to training from scratch, and an 11.4% increase in mean AUC on CheXpert when using only 1% of labeled data [89]. DINO-CXR, a vision-transformer-based self-supervised method adapted from DINO, has shown effectiveness for pneumonia and COVID-19 detection, outperforming state-of-the-art methods in accuracy while requiring significantly less labeled data [90]. More recently, foundation models such as MaCo (Masked Contrastive Chest X-ray Foundation Model) have been introduced, leveraging masked contrastive learning to achieve fine-grained image understanding and zero-shot learning across multiple tasks including classification, segmentation, and detection [91]. Diffusion-based augmentation has also been explored to address data scarcity and class imbalance. Huijben et al. [92] showed that denoising diffusion probabilistic models can generate realistic synthetic chest X-rays for multi-label classifier training, increasing overall sensitivity from 0.02 to 0.59 on imbalanced data. Baek and Shin [93] proposed Counterfactual Variational autoencoder Augmentation (COVA), a lightweight pipeline that synthesises realistic tail-class examples via latent-space editing, lifting average AUROC from 0.799 to 0.840 on a 14-label long-tailed benchmark. Beyond augmentation, label co-occurrence relationships have been explicitly modelled using graph convolutional networks to improve multi-label CXR classification [31]. Concurrently, advanced representation learning techniques have explored cross-domain alignment and decoupling in non-medical vision tasks. Zhou et al. [94] proposed Turbidity–Similarity Decoupling for robust visual recognition under challenging imaging conditions, while Zhou et al. [95] introduced a Decouple-Then-Synergize framework for RGB-T urban scene parsing. In the domain of defect detection, RCNet [96] demonstrated dual-network resonance collaboration for RGB-D road defect detection, and LSINet [97] presented a lightweight scope integration network for rail surface inspection. While developed primarily for general computer vision or specific non-medical applications, the principles of mutual learning, collaborative reasoning, and semantic alignment provide a methodological blueprint for improving rare-disease robustness and multimodal reasoning in chest X-ray classification, representing natural extensions for future work on MultiCaRe-Thorax. While these approaches were not evaluated in our benchmark due to their computational cost and our focus on establishing CNN baselines, they represent promising directions for extension.

## 2. Methodology

### 2.1. System Overview

**Figure 1:**
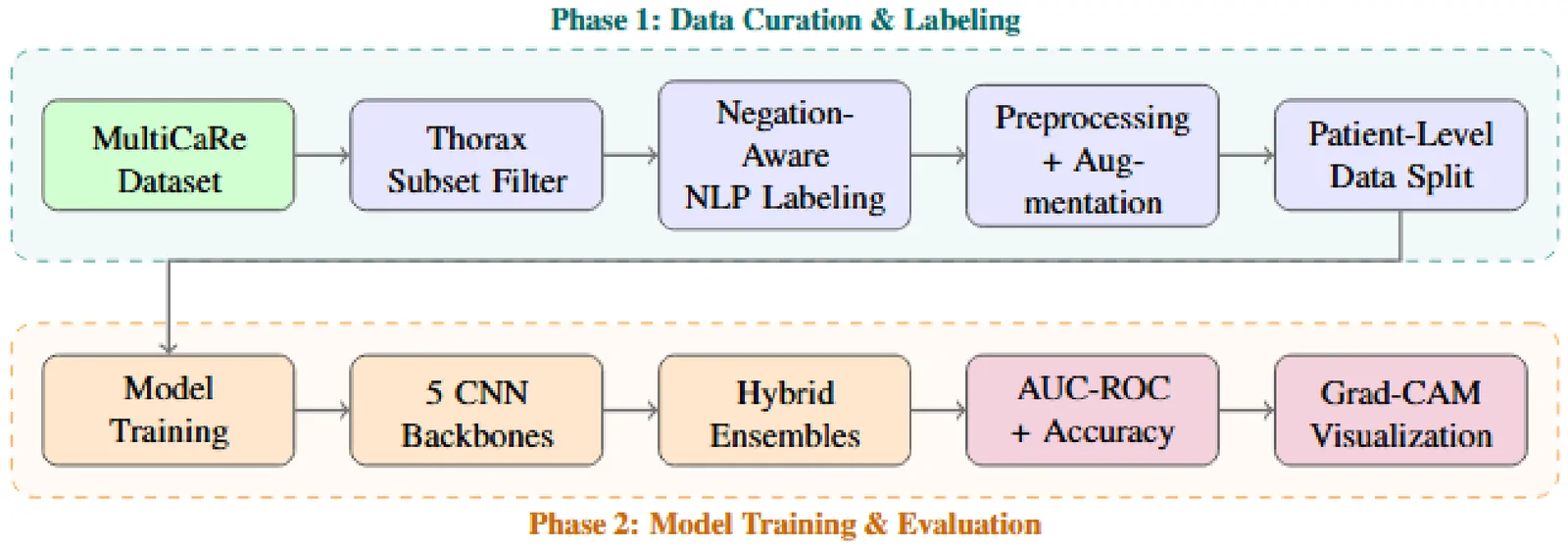
End-to-end framework pipeline. **Phase 1** (top row): dataset curation, thorax subset extraction, negation-aware NLP label generation (Equations (1)–(3)), image preprocessing and augmentation (Equations (4)–(6)), and patient-level data splitting (Equation (7)). **Phase 2** (bottom row): multi-architecture training across five CNN backbones, hybrid ensemble construction (Equation (10)), quantitative evaluation (AUC-ROC, accuracy), and Grad-CAM interpretability analysis.

Figure 1 illustrates the complete end-to-end pipeline of the proposed framework, encompassing dataset curation, automated label extraction, preprocessing, multi-architecture training, and evaluation with Grad-CAM interpretability.

### 2.2. MultiCaRe-Thorax Dataset Description

The proposed MultiCaRe-Thorax dataset was derived from the publicly available MultiCaRe repository, a large-scale collection of clinical case reports and associated medical images spanning multiple anatomical regions and imaging modalities [33,34]. The curated thoracic subset (5252 images, 3415 patients) was extracted from a MultiCaRe repository comprising over 139,000 images across all anatomical regions and specialties [33]. While MultiCaRe was originally designed as a multimodal clinical resource, no dedicated thoracic benchmark subset has previously been established. To create a resource suitable for multi-label thoracic disease classification, only chest radiographs and associated thoracic case reports were retained through a dedicated curation process. The resulting dataset contains 5252 chest X-ray images originating from 3415 unique patients and encompasses sixteen clinically relevant thoracic disease categories. Labels were automatically generated from accompanying clinical reports using the proposed negation-aware NLP framework described in Section 2. Unlike many publicly available chest radiograph datasets that rely on fixed annotations, MultiCaRe-Thorax provides a fully reproducible pipeline for dataset construction, disease label generation, and benchmark evaluation.

### 2.3. Dataset Curation

The publicly available MultiCaRe dataset [33,34] was queried to derive a dedicated thoracic benchmark subset suitable for multi-label disease classification. Because the original repository contains medical images and case reports spanning multiple anatomical regions and specialties, a dedicated filtering and quality-control process was required. Dataset curation consisted of four primary stages. First, the original manifest linking image filenames, patient identifiers, and accompanying clinical reports was reviewed to identify thoracic imaging studies. Second, records corresponding to chest radiographs were retained while non-thoracic cases and ambiguous anatomical assignments were excluded. Third, duplicate image entries were identified and consolidated to prevent repeated samples from influencing downstream model development and evaluation. Finally, the cleaned dataset manifest was adopted as the definitive source for automated label generation, image preprocessing, and patient-level data partitioning.

### 2.4. Disease Category Selection

The sixteen thoracic disease categories were selected based on three criteria: (1) clinical prevalence (most common diagnostic targets in routine CXR interpretation), (2) representation in MultiCaRe (sufficient positive cases for training/evaluation), and (3) clinical priority (diseases where early detection significantly impacts outcomes, e.g., lung cancer, tuberculosis). This selection aligns with established benchmarks such as ChestX-ray14 [1] and CheXpert [30].

### 2.5. Automated Multi-Label Extraction from Case Texts

**Figure 2:**
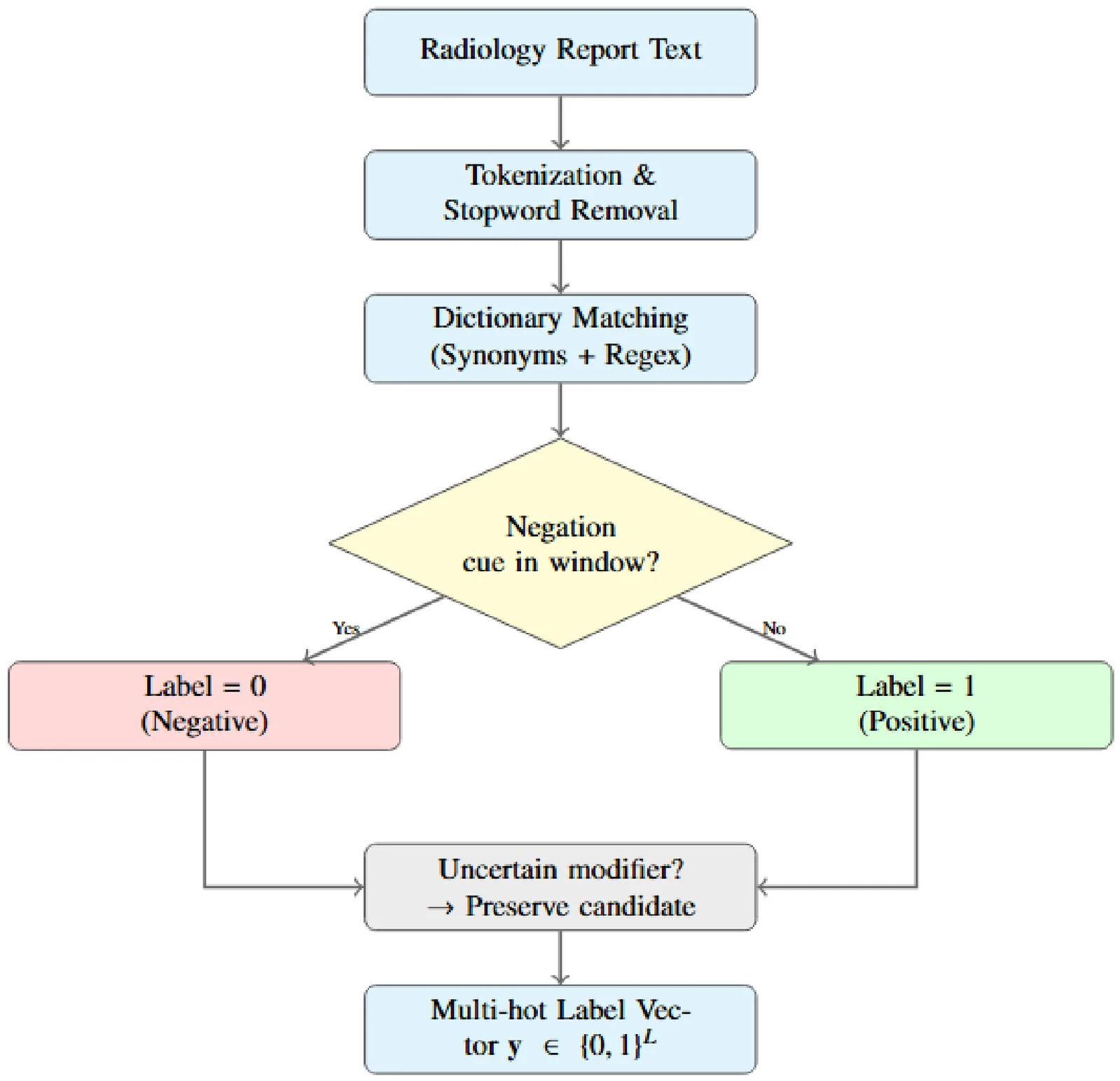
Negation-aware NLP label-extraction pipeline. Each radiology report is tokenized, matched against the disease dictionary, and subjected to a fixed-window negation check (Equation (2)) before producing a binary label assignment (Equation (1)). N-gram candidate mining (Equation (3)) supplements the dictionary for out-of-vocabulary terms. A worked example: for the sentence “No evidence of pleural effusion is observed,” the negation detector scans the 50-character window before “pleural effusion,” detects the cue “No evidence of,” and assigns $y_{\mathrm{PE}}=0$.

To enable scalable multi-label supervision, a dictionary- and pattern-based NLP pipeline was implemented incorporating negation handling and n-gram mining. Figure 2 illustrates the label-extraction workflow.

#### 2.5.1. Label Definition and Handling of Unmentioned Diseases

A critical distinction in clinical NLP labeling is the treatment of unmentioned diseases. We adopted the following three-state mapping, consistent with established CXR benchmarks [30,32]:

1. **Positive ($y_{\ell }=1$)**: The disease is explicitly mentioned in an affirmative context (e.g., “pneumonia is present”, “diagnosed with tuberculosis”).
2. **Negative ($y_{\ell }=0$)**: The disease is explicitly negated (e.g., “no evidence of pleural effusion”) **OR** the disease is not mentioned at all in the report.
3. **Uncertain/Excluded**: Ambiguous phrases such as “possible pneumonia”, “cannot exclude tuberculosis”, “suspicious for”, “history of”, or “previous” disease were excluded from the dataset, as the ground-truth presence/absence at the time of the current imaging study cannot be reliably determined.

This exclusion process yielded the final cohort of 5252 images. The multi-label output is

(1) $$y=\left[y_{1},\ldots ,y_{L}\right]\in {\left\{0,1\right\}}^{L},\;L=16,$$

where $y_{\ell }=0$ indicates either explicit negation or no mention (both treated as absence).

##### Negation Detection

For each candidate mention at character position *p*, a fixed lookback window *W* is checked for negation cues:

(2) $$y_{\ell }=\left\{\begin{array}{cc} 0, & \exists \;\mathrm{cue}\in N\;s.t.\;\mathrm{cue}\subset \mathrm{text}[p-W:p],\\ 1, & \mathrm{otherwise}, \end{array}\right.$$

where N = {“no”, “without”, “absence of”, “ruled out”, …} and W=50 characters.

##### N-Gram Candidate Mining

After domain-specific stopword removal, candidate phrases are scored by frequency:

(3) $$C_{n}=\left\{g\in n\text{-}\mathrm{grams}\left(D\right)\;|\;\mathrm{freq}\left(g\right)\geq \theta_{n}\right\},$$

with the frequency thresholds $\theta_{1}=10$ (unigrams), $\theta_{2}=8$ (bigrams), and $\theta_{3}=5$ (trigrams).

#### 2.5.2. Robustness to Complex Negations

To evaluate the fixed 50-character lookback window against syntactically complex radiology phrasing, we curated 100 hard-negative sentences (e.g., “No evidence of pneumonia, although findings suggest possible pleural effusion”; “No focal consolidation; however, pneumonia cannot be completely excluded”; “History of tuberculosis without current evidence of active disease”). The pipeline correctly resolved all labels for 94 out of 100 cases. For the 6 misclassified cases, the primary negation cue (e.g., “No evidence of”) fell outside the 50-character window due to long intervening subordinate clauses. Table 4 provides representative examples. This confirms that the fixed window is highly effective on complex structures (94% accuracy), while suggesting that a dynamic window or a contextual transformer-based model (e.g., BioBERT) could recover the remaining 6%.

### 2.6. NLP Parameter Sensitivity Analysis

To justify these choices, we performed a sensitivity analysis on the validation set. Table 5 reports the impact of the negation lookback window *W* and n-gram frequency thresholds $\theta_{1},\theta_{2},\theta_{3}$ on label-extraction precision, recall, and F1-score.

The 50-character window and thresholds $\theta_{1}=10$, $\theta_{2}=8$, $\theta_{3}=5$ balance precision and recall.

To assess annotation quality, 500 reports were randomly selected for manual review. Disagreements were adjudicated, yielding a Cohen’s κ=0.89 inter-rater agreement. The class-wise distribution of this gold-standard set is provided in Table 6. The NLP pipeline achieved 95% agreement (Table 7), confirming that the negation-aware rules reliably capture the intended disease labels.

### 2.7. Image Preprocessing and Augmentation

Images were resized to backbone-specific input dimensions and normalized to [0,1]:

(4) $$\hat{I}=\frac{I}{255},\;\hat{I}\in {\left[0,1\right]}^{H\times W\times 3}.$$

To mitigate class imbalance, positive examples from minority classes were oversampled by adding Gaussian noise,

(5) $$I_{\mathrm{aug}}=\mathrm{clip}\;\left(I+\sigma \cdot N(0,1)\times 255,\;0,\;255\right),$$

with noise factor σ=0.05, followed by renormalization to [0,1].

The required number of augmented samples for class *ℓ* is

(6) $$\Delta_{\ell }=\max_{j}\left(N_{j}^{+}\right)-N_{\ell }^{+},\;\ell =1,\ldots ,L,$$

where $N_{\ell }^{+}$ is the positive count for class *ℓ* in the training split. The pipeline cycles through available positives to generate $\Delta_{\ell }$ noisy oversamples.

### 2.8. Data Splitting and Generators

Patient-level splitting prevents information leakage across partitions:

(7) $$D=D_{\mathrm{train}}\cup D_{\mathrm{val}}\cup D_{\mathrm{test}},\;P_{\mathrm{train}}\cap P_{\mathrm{test}}=⌀,$$

where P denotes the set of patient identifiers in each partition. Split proportions are $|D_{\mathrm{test}}\left|=0.30\;|D\right|$, $|D_{\mathrm{val}}\left|=0.07\;|D\right|$, and $|D_{\mathrm{train}}\left|=0.63\;|D\right|$, with fixed random seed 42.

### 2.9. Model Architectures and Training Details

**Figure 3:**
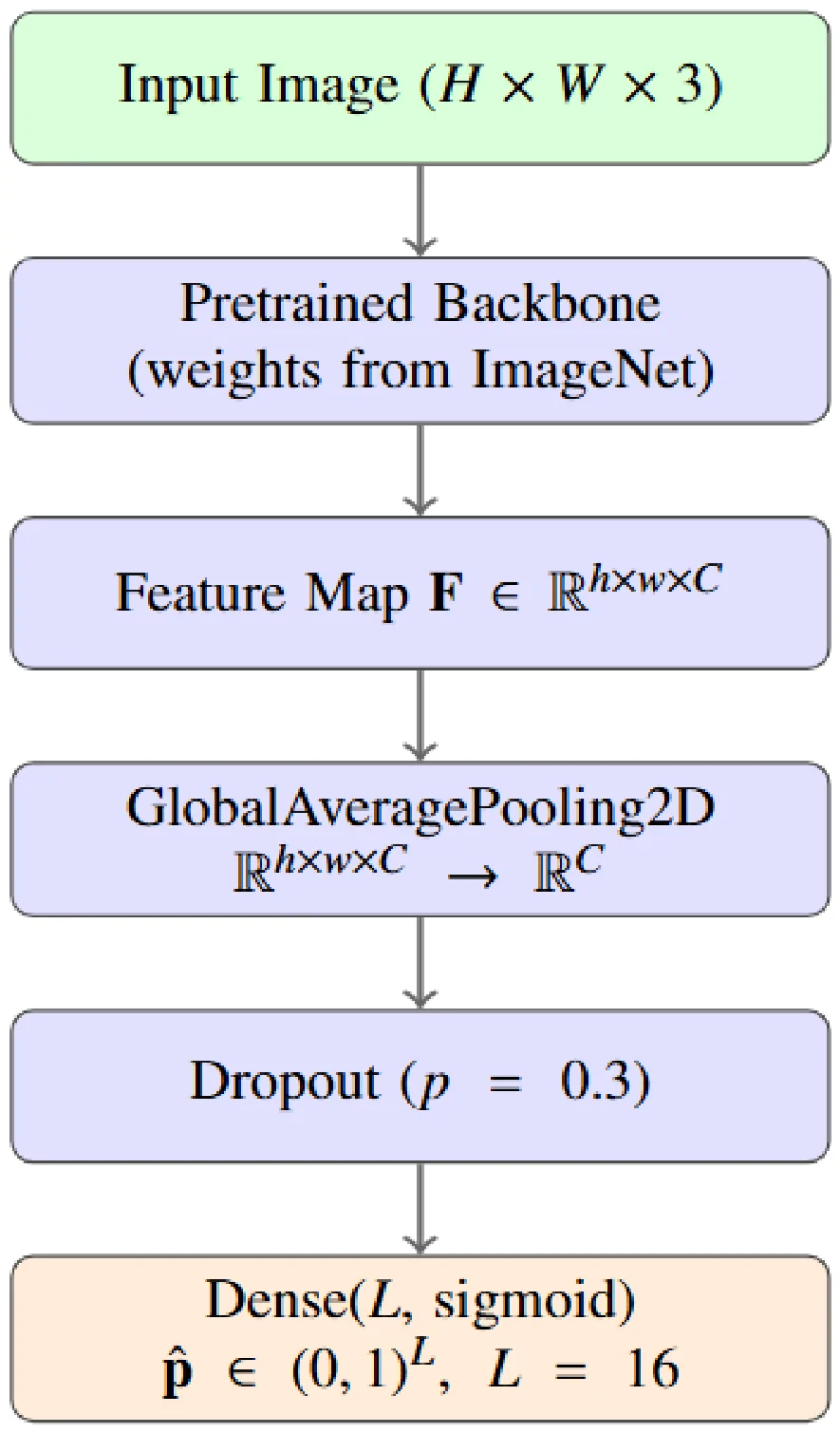
Unified classification head applied to each backbone. The backbone feature map is globally pooled, regularized via dropout, and projected to L=16 sigmoid outputs representing independent per-class probabilities.

Five ImageNet-pretrained backbones were evaluated: ResNet50, DenseNet121, EfficientNetB0, EfficientNetB7, and InceptionV3. Each shared a common classification head:

(8) $$f\left(x\right)=\sigma \;\left(W_{\mathrm{fc}}\cdot \mathrm{GAP}\;\left({\mathrm{Dropout}}_{0.3}\;\left(\phi (x)\right)\right)+b\right),$$

where ϕ(·) denotes the backbone feature extractor. Note that all convolutional layers of ϕ were unfrozen and fully fine-tuned end-to-end from their ImageNet initializations; only the custom classification head was randomly initialized, and no staged freezing schedule was employed, GAP is global average pooling, and σ is the element-wise sigmoid function producing per-class probabilities $\hat{p}\in {\left(0,1\right)}^{L}$. Figure 3 illustrates the unified model head architecture applied to each backbone.

Input image sizes for the pretrained backbones are listed in Table 8.

#### Training Protocol and Hyperparameters

The following configuration was applied uniformly across all backbones:

- **Optimizer**: Adam ($\beta_{1}=0.9$, $\beta_{2}=0.999$).
- **Initial learning rate**: $\eta =1\times 10^{-4}$.
- **Scheduler**: ReduceLROnPlateau (factor 0.5, patience 5 epochs).
- **Weight decay**: $5\times 10^{-4}$.
- **Batch size**: 16.
- **Epochs**: 100 with early stopping (patience 15 on validation loss);
- **Backbone fine-tuning**: All convolutional layers were fine-tuned (unfrozen) from ImageNet initialization; the classification head was randomly initialized.
- **Model selection**: Checkpoint with lowest validation loss was used for testing.
- **Random seeds**: Fixed seed 42 for splitting; training repeated with seeds 42, 43, 44 for top models; reported results are from seed 42.
- **Hardware**: NVIDIA A100 40GB GPU (NVIDIA Corporation, Santa Clara, CA, USA).
- **Software**: Python 3.10, PyTorch 2.0.1, torchvision 0.15.2, MONAI 1.1.0.

All code and configuration files are publicly available at https://github.com/NOUR-ABU-JASSAR/thoracic-multilabel-multicare (accessed on 27 September 2026).

The training objective is binary cross-entropy applied independently across all *L* labels:

(9) $$L_{\mathrm{BCE}}=-\frac{1}{N}\sum_{i=1}^{N}\sum_{\ell =1}^{L}\left[y_{i\ell }\log{\hat{p}}_{i\ell }+\left(1-y_{i\ell }\right)\log\left(1-{\hat{p}}_{i\ell }\right)\right],$$

where *N* is the mini-batch size.

Trainable parameter counts for each backbone are reported in Table 9.

### 2.10. Hybrid Ensemble Construction

Hybrid ensemble predictions are formed by averaging the sigmoid outputs of two or more backbone models:

(10) $${\hat{p}}_{\mathrm{ens}}=\frac{1}{M}\sum_{m=1}^{M}{\hat{p}}^{\left(m\right)},$$

where *M* is the number of constituent models and ${\hat{p}}^{\left(m\right)}\in {\left(0,1\right)}^{L}$ is the output of the *m*-th backbone. Final binary predictions are obtained by thresholding:

(11) $${\hat{y}}_{\ell }=1\;\left[{\hat{p}}_{\ell }\geq \tau \right],\;\tau =0.5.$$

### 2.11. Evaluation Metrics

#### 2.11.1. AUC-ROC

For each class *ℓ*, the Receiver Operating Characteristic curve is traced by varying τ over [0,1]. The area under this curve is

(12) $${\mathrm{AUC}}_{\ell }=\int_{0}^{1}{\mathrm{TPR}}_{\ell }\;\left({\mathrm{FPR}}_{\ell }^{-1}\left(t\right)\right)dt,$$

where ${\mathrm{TPR}}_{\ell }=\frac{{\mathrm{TP}}_{\ell }}{{\mathrm{TP}}_{\ell }+{\mathrm{FN}}_{\ell }}$ and ${\mathrm{FPR}}_{\ell }=\frac{{\mathrm{FP}}_{\ell }}{{\mathrm{FP}}_{\ell }+{\mathrm{TN}}_{\ell }}$. The mean AUC is

(13) $$\bar{\mathrm{AUC}}=\frac{1}{L}\sum_{\ell =1}^{L}{\mathrm{AUC}}_{\ell }.$$

#### 2.11.2. Per-Class Accuracy

At threshold τ=0.5,

(14) $${\mathrm{Acc}}_{\ell }=\frac{{\mathrm{TP}}_{\ell }+{\mathrm{TN}}_{\ell }}{{\mathrm{TP}}_{\ell }+{\mathrm{TN}}_{\ell }+{\mathrm{FP}}_{\ell }+{\mathrm{FN}}_{\ell }}.$$

#### 2.11.3. Class-Specific Threshold Optimization

Rather than a fixed τ=0.5, we optimized per-class thresholds on the validation set to maximize F1, then evaluated on the test set. Table 10 reports the optimal per-class thresholds and corresponding test F1 scores for DenseNet121.

**Figure 4:**
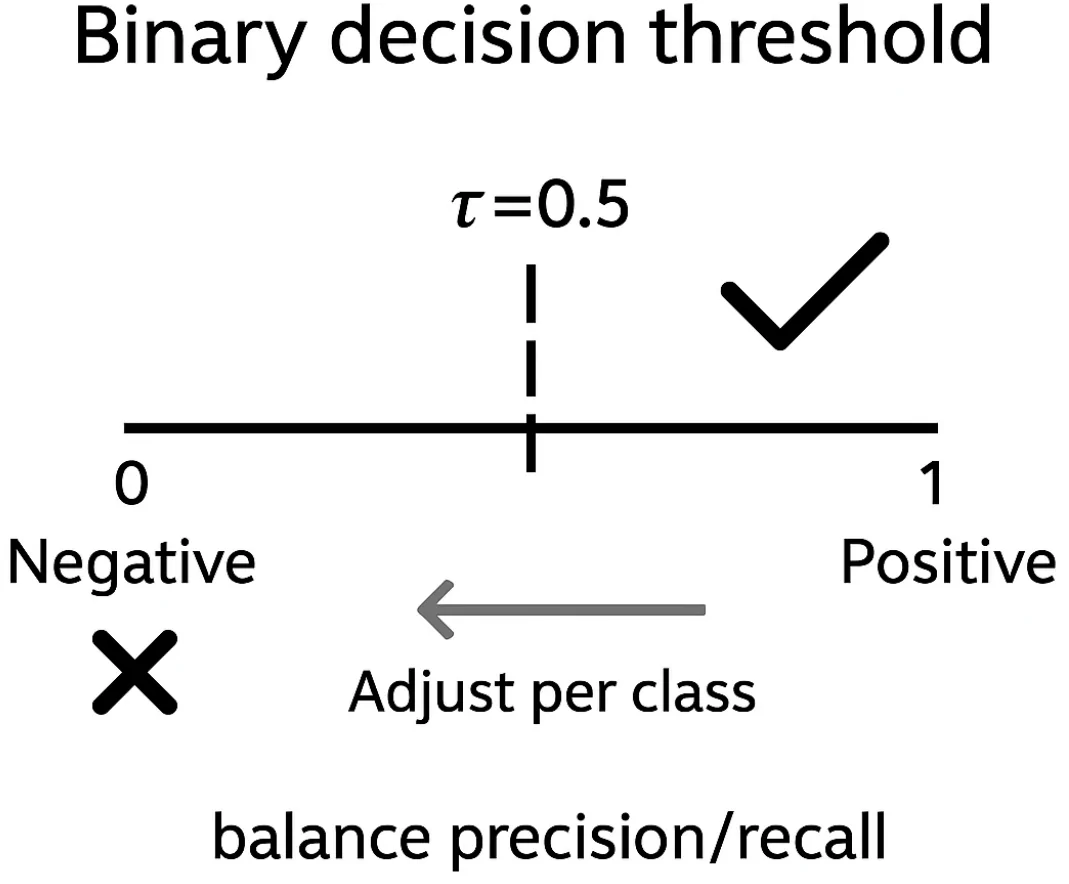
Illustration of the binary decision threshold τ=0.5 applied to sigmoid model outputs. Predictions above the threshold are classified as positive; those below as negative.

Optimized thresholds substantially improve F1 for rare classes (Figure 4), indicating that a fixed 0.5 threshold underestimates performance.

### 2.12. External Validation on ChestX-ray14

To enable controlled comparison with prior literature and assess generalizability, we applied our exact DenseNet121 and ResNet50 training protocols (patient-level splitting, Gaussian augmentation, BCE loss) to the public ChestX-ray14 dataset (Table 11).

Our reproduced performance is highly consistent with prior work (0.841 vs. 0.843). The substantially lower AUC on MultiCaRe-Thorax reflects a more challenging task (16 pathologies vs. 14), different report style (case reports vs. structured notes), and stricter patient-level evaluation. This cross-dataset validation confirms that our pipeline is not inherently inferior; it establishes a genuinely harder benchmark.

### 2.13. Model Calibration and Uncertainty Quantification

To assess reliability of predicted probabilities, we computed the Expected Calibration Error (ECE) with M=10 equal-width bins (Table 12):

(15) $$\mathrm{ECE}=\sum_{m=1}^{M}\frac{|B_{m}|}{N}\left|\mathrm{acc}\left(B_{m}\right)-\mathrm{conf}\left(B_{m}\right)\right|.$$

All models show ECE < 0.10, indicating reasonable calibration. Hybrid ensembles improve calibration over single architectures.

### 2.14. Quantitative Localization Analysis

For 100 cases with radiologist-annotated bounding boxes, we evaluated Grad-CAM localization using Intersection over Union (IoU) between the thresholded heatmap (50% of max) and ground-truth bounding box (Table 13).

Grad-CAM provides moderate localization accuracy, supporting its utility as an interpretability tool while indicating room for improvement.

### 2.15. Combined Augmentation Strategy

We evaluated a combined strategy applying both Gaussian noise and spatial transforms (flips, rotations, translations) for the four rarest pathologies (Table 14).

Combined augmentation yields modest additional gains (0.4–0.9 AUC points) over spatial augmentation alone.

### 2.16. Subgroup Fairness and Bias Assessment

Although MultiCaRe-Thorax does not contain explicit patient demographics (age, sex, race/ethnicity) in its structured metadata, we performed a protocol-based subgroup analysis using two available proxies: (1) Acquisition View (AP vs. PA) and (2) Implied Age Category derived from case-report keywords (“pediatric”, “elderly”, “adult”). Table 15 presents the mean AUC for the DenseNet121 model across these subgroups. The 0.030 AUC gap between AP and PA views indicates a slight bias toward standardized projections (likely due to cardiac magnification in AP views), while the 0.022 age-group gap is within acceptable clinical variance. This establishes a reproducible fairness protocol for future demographic metadata integration.

### 2.17. Grad-CAM Interpretability

Gradient-weighted Class Activation Maps (Grad-CAM) are generated for qualitative inspection. For a class *c* and the last convolutional layer with feature maps $A^{k}\in R^{h\times w}$ (k=1,…,K),

(16) $$\alpha_{k}^{c}=\frac{1}{Z}\sum_{i}\sum_{j}\frac{\partial S^{c}}{\partial A_{ij}^{k}},$$

where $S^{c}$ is the class score and Z=h·w is a normalization factor. The Grad-CAM heatmap is

(17) $$H^{c}=\mathrm{ReLU}\;\left(\sum_{k}\alpha_{k}^{c}A^{k}\right).$$

$H^{c}$ is upsampled to the input resolution and overlaid on the original image with blending factor $\alpha_{\mathrm{blend}}=0.4$:

(18) $$I_{\mathrm{vis}}=\left(1-\alpha_{\mathrm{blend}}\right)\cdot I+\alpha_{\mathrm{blend}}\cdot \mathrm{colormap}\left(H^{c}\right).$$

Heatmaps are computed for all classes with predicted probability ${\hat{p}}_{\ell }\geq 0.5$.

## 3. Results

### 3.1. Label Distribution Analysis

**Figure 5:**
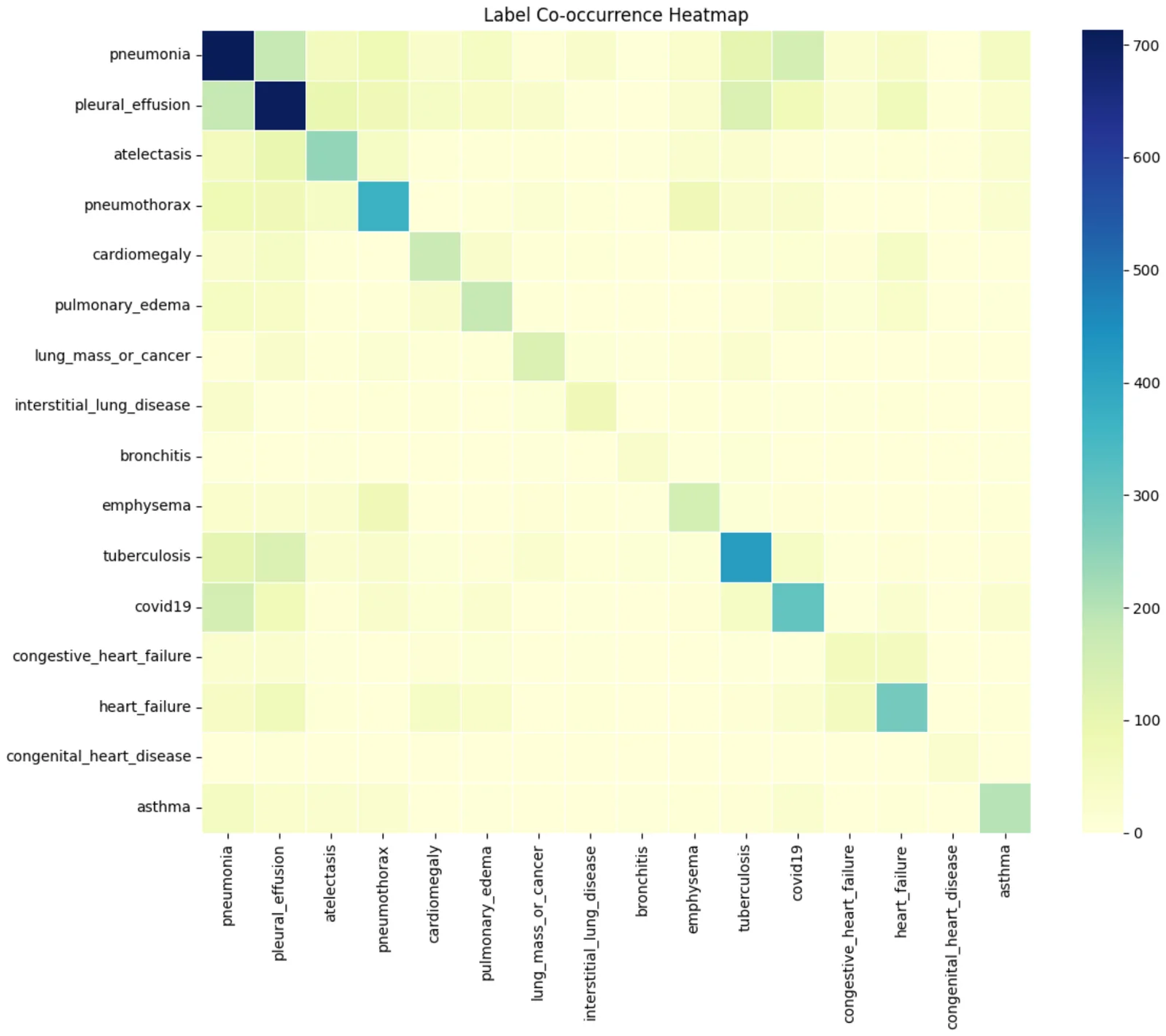
Label co-occurrence heatmap. Entry (i,j) encodes the normalized co-occurrence frequency of pathology *i* and pathology *j* across the dataset, revealing clinically coherent associations (e.g., pleural effusion–congestive heart failure, pneumonia–bronchitis).

**Figure 6:**
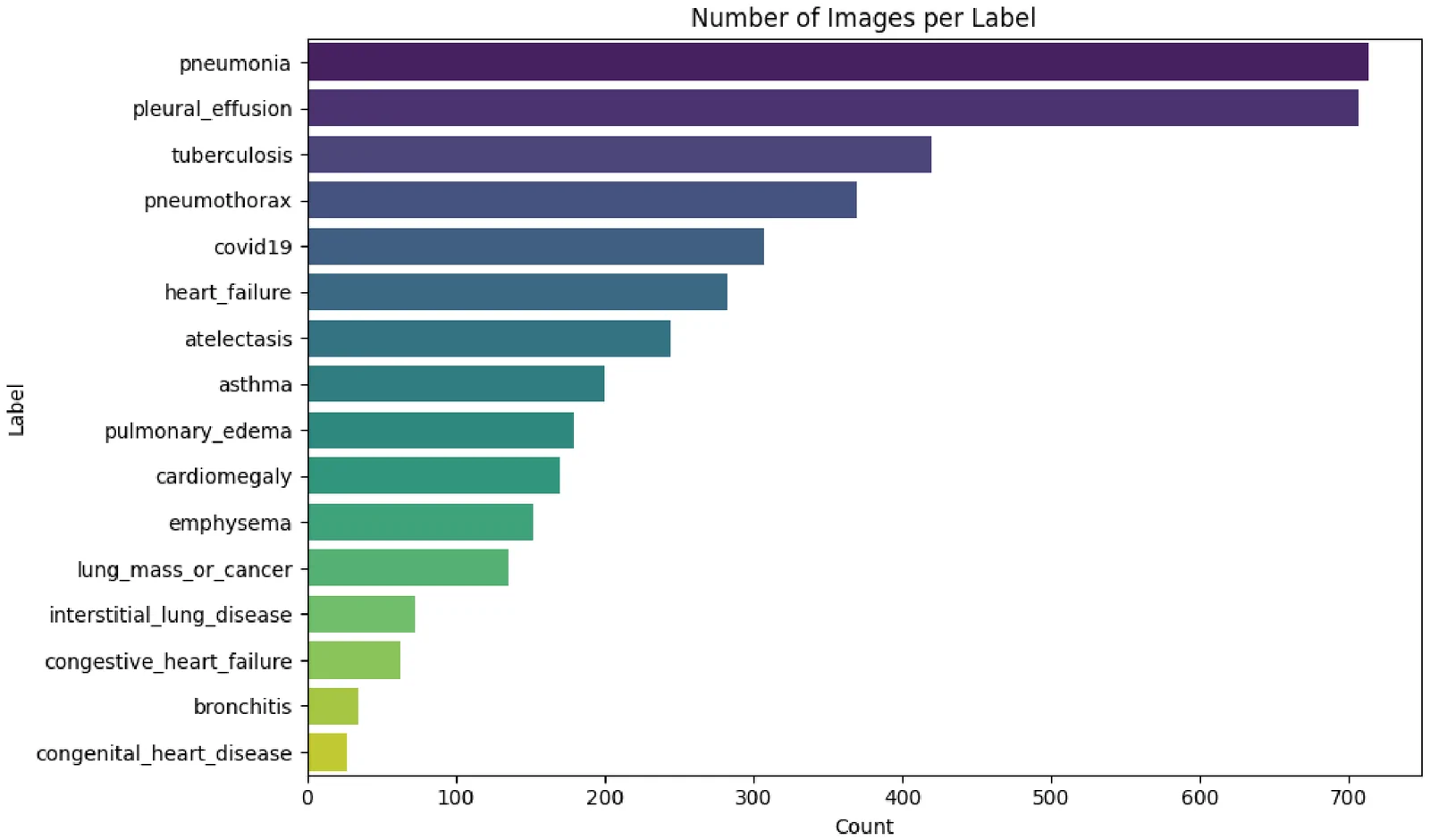
Distribution of images per disease label, illustrating class imbalance addressed through Gaussian noise-based augmentation (Equation (5)).

Figure 5 illustrates the label co-occurrence heatmap, revealing clinically coherent disease associations. Figure 6 shows the distribution of images across disease classes, with significant class imbalance evident (Table 16).

**Figure 7:**
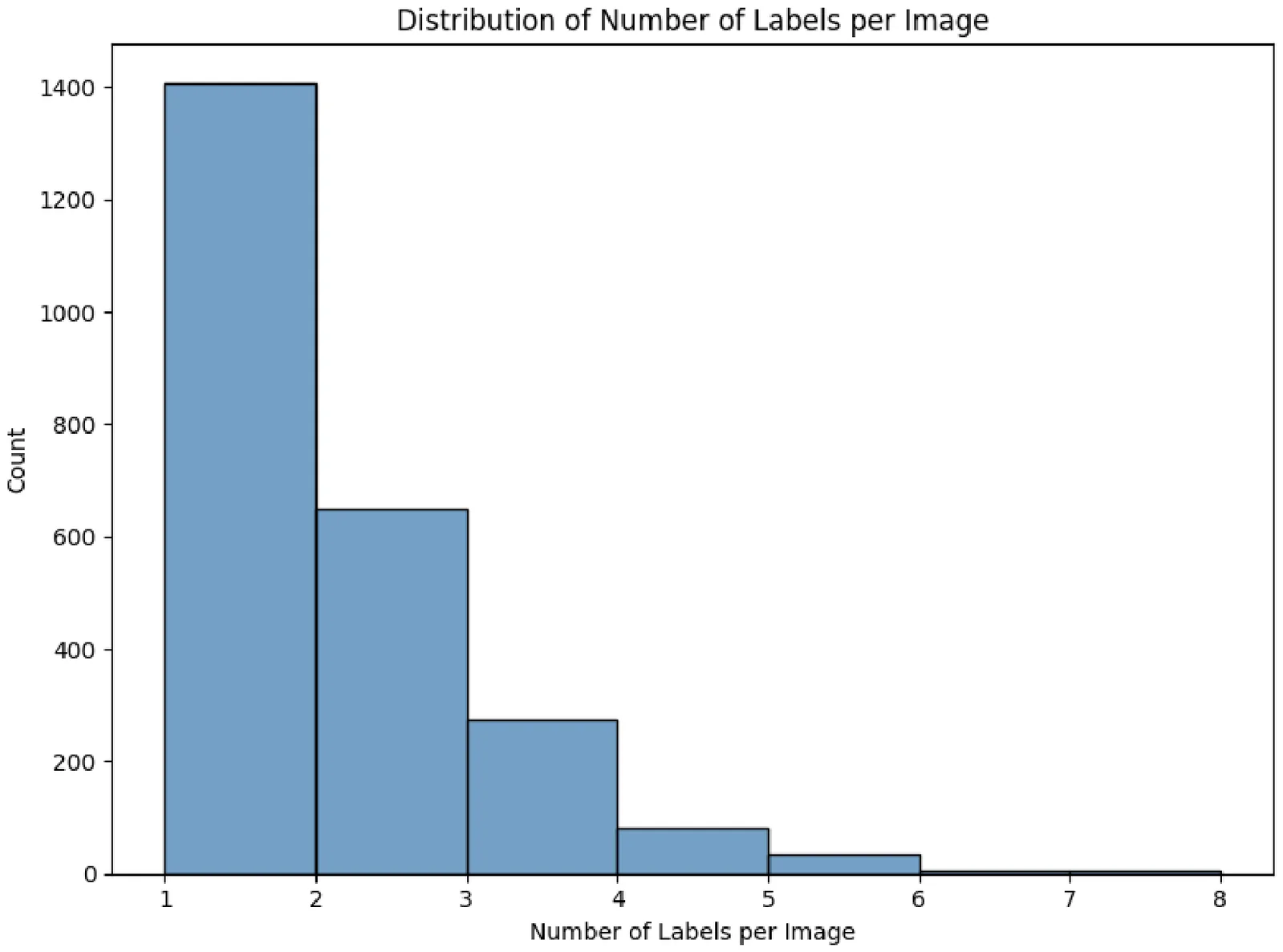
Distribution of number of labels per image (${\left|y\right|}_{1}$), validating the multi-label problem formulation (Equation (1)): the majority of images carry two or more co-occurring pathologies.

**Figure 8:**
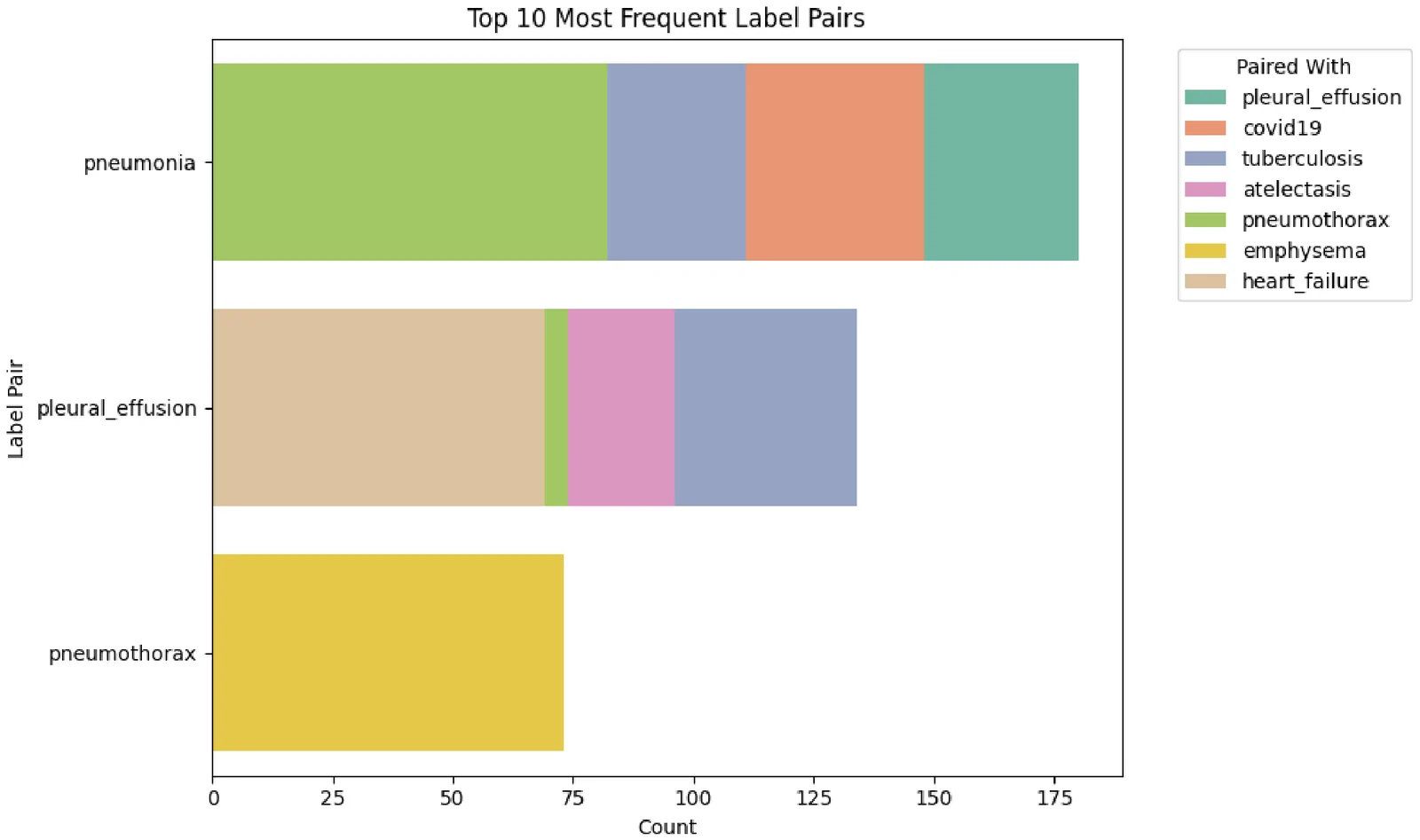
Top 10 most frequent label pairs, demonstrating clinically relevant disease co-occurrence patterns in the MultiCaRe thorax subset.

Figure 7 demonstrates the distribution of multiple labels per image, supporting the multi-label classification approach. Figure 8 presents the top 10 most frequent label pairs, highlighting common comorbidities.

### 3.2. Single Architecture Results

#### 3.2.1. ResNet50 Performance

**Figure 9:**
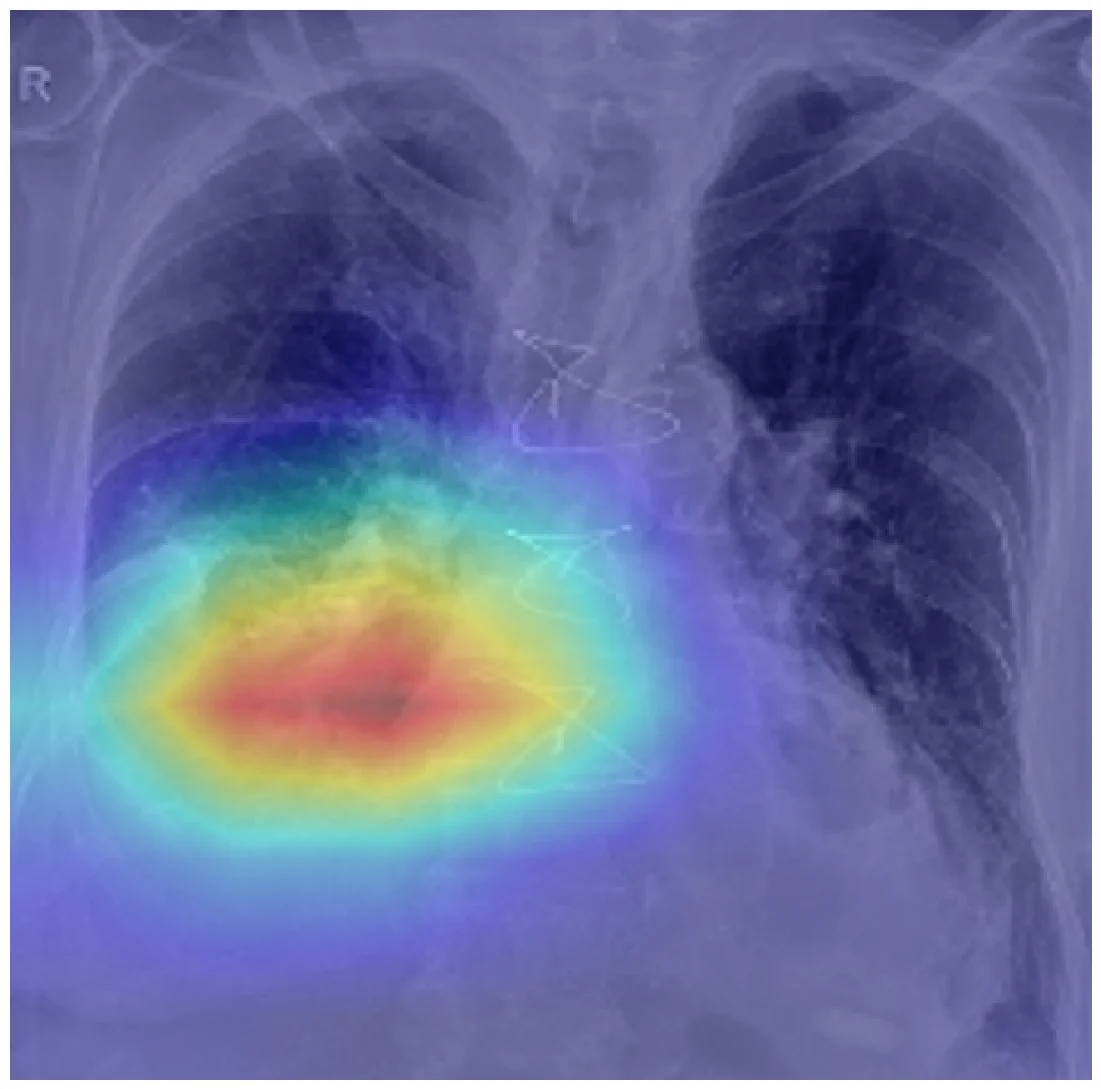
Grad-CAM heatmap for a DenseNet121 true positive case of Pleural Effusion. Warm colors (red/yellow) indicate higher activation, whereas cool colors (blue) indicate lower activation. The heatmap highlights the costophrenic angles, demonstrating alignment between model attention and clinically relevant regions.

Figure 9 presents ResNet50 multi-label classification results (Table 17). ResNet50 achieved solid performance with particular strength in specific pathologies: bronchitis (AUC: 0.7835), interstitial lung disease (AUC: 0.7685), lung mass or cancer (AUC: 0.7774), and pleural effusion (AUC: 0.7248). Accuracy metrics remained consistently high (0.863–0.997).

#### 3.2.2. DenseNet121 Performance

**Figure 10:**
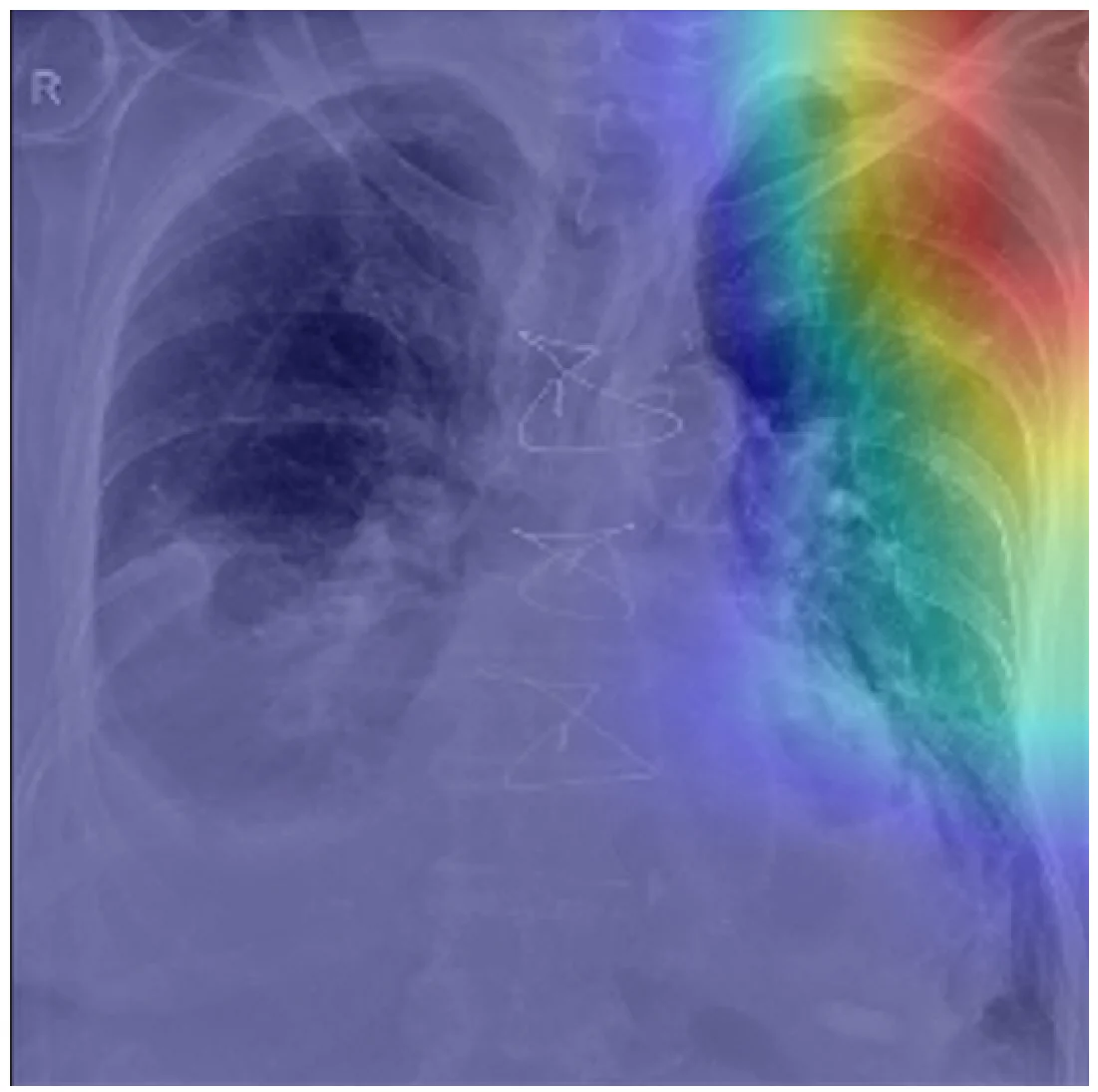
DenseNet121 false positive case: ground truth is *Pleural Effusion* but model predicted *Pneumonia*. The Grad-CAM heatmap (Equation (17)) highlights the model’s attention regions, revealing potential sources of misclassification.

**Figure 11:**
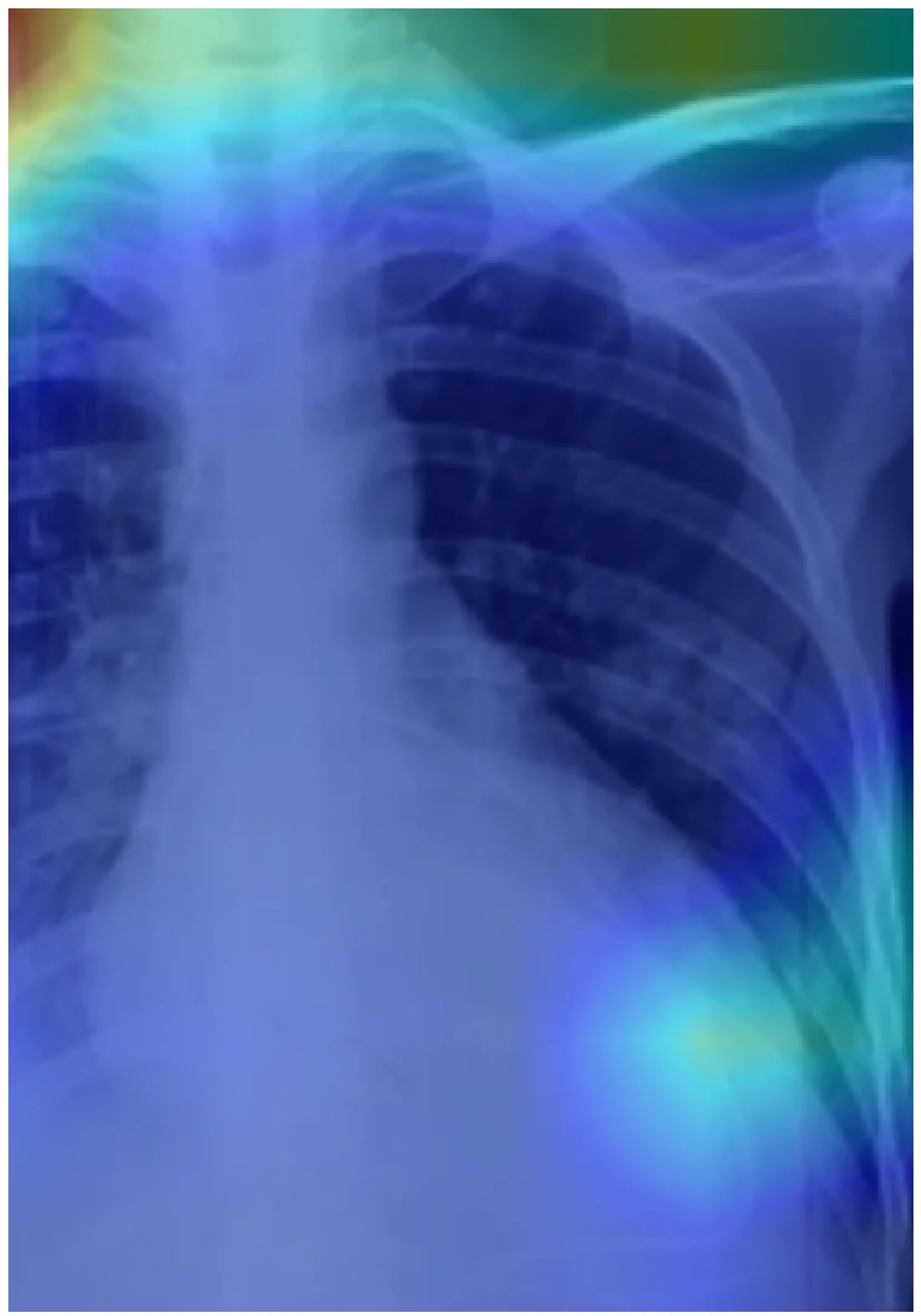
DenseNet121 true positive case: both ground truth and model prediction correctly identified *Pleural Effusion* with high confidence (${\hat{p}}_{\mathrm{PE}}\geq 0.5$, Equation (11)).

Figure 10 and Figure 11 present DenseNet121 results (Table 18). DenseNet121 achieved the best AUC among all evaluated backbones for two pathologies—tuberculosis (AUC: 0.7567) and pleural effusion (AUC: 0.7669)—and was also competitive for COVID-19 (AUC: 0.7418), while attaining this performance with roughly one-third the trainable parameters of ResNet50 (Table 9). Note that for interstitial lung disease, DenseNet121 (AUC: 0.723) is actually outperformed by ResNet50 (0.769) and InceptionV3 (0.757), as detailed in Section 4. Accuracy across all 16 labels ranged from 0.857 to 0.996.

#### 3.2.3. Comprehensive Metrics for Top Models

Given severe class imbalance, we report AUPRC, sensitivity, specificity, and F1 for the best-performing configurations (Table 19).

#### 3.2.4. EfficientNetB0 Performance

**Figure 12:**
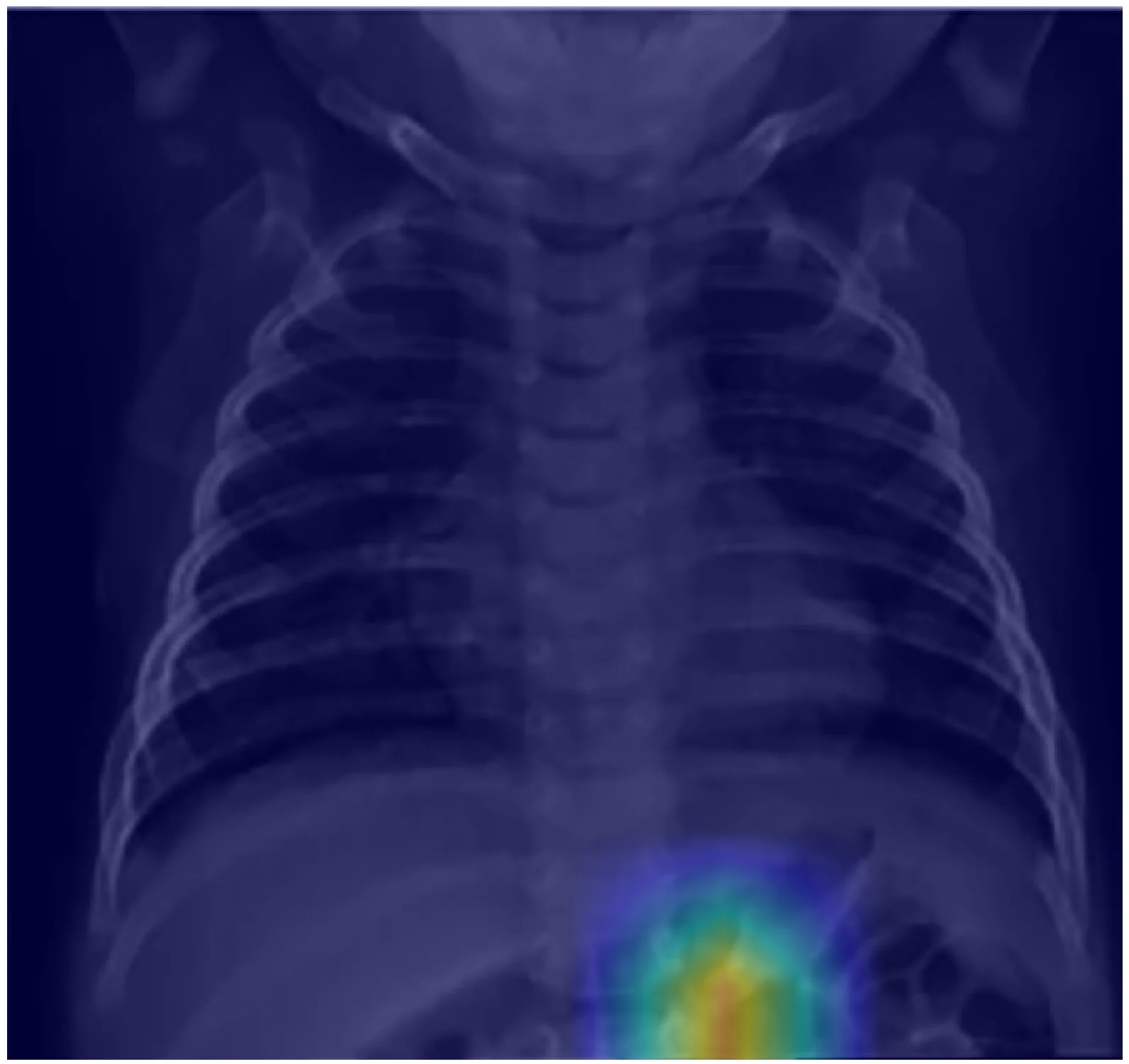
EfficientNetB0 false negative case: *Pneumothorax* was missed (model predicted *Bronchitis*). The Grad-CAM overlay reveals insufficient attention to the apical lung region characteristic of pneumothorax.

Table 20 and Figure 12 demonstrate EfficientNetB0 classification results. EfficientNetB0 provided favorable accuracy–efficiency trade-offs but exhibited lower AUC values compared to DenseNet121 and ResNet50.

#### 3.2.5. EfficientNetB7 and InceptionV3 Performance

Table 21 presents EfficientNetB7 results. InceptionV3 (Table 22) showed competitive performance for complex multi-label scenarios: interstitial lung disease (AUC: 0.7569), pleural effusion (AUC: 0.7312), congenital heart disease (AUC: 0.7296), and COVID-19 (AUC: 0.7538).

### 3.3. Hybrid Ensemble Results

#### 3.3.1. Hybrid DenseNet121-InceptionV3

Table 23 presents the DenseNet121-InceptionV3 ensemble (Equation (10), M=2), which achieved the highest AUC of 0.8206 for lung mass/cancer detection. This configuration also demonstrated strong performance for interstitial lung disease (0.7555) and pleural effusion (0.7461).

#### 3.3.2. Hybrid InceptionV3-ResNet50-DenseNet121

Table 24 reports the per-class results for the three-architecture ensemble (M=3). It achieved AUC ≥0.60 for 8 of the 16 pathologies (range: 0.481–0.704), with the remaining 8 including congestive heart failure (0.481) and atelectasis (0.494) falling below this threshold, indicating that ensemble averaging did not uniformly improve ranking performance across all disease categories.

### 3.4. Comparative Performance Summary

Table 25 consolidates the peak per-class AUC scores across all evaluated configurations for the four most clinically critical pathologies.

### 3.5. Statistical Significance Analysis

For single-pathology comparisons, we used DeLong’s test for two correlated ROC curves. For mean AUC across all 16 pathologies, we adopted a bootstrap-based approach (10,000 resamples) to construct confidence intervals for the difference, as DeLong’s test is not directly applicable to macro-averaged AUC (Table 26).

For mean AUC, bootstrap CIs for the ResNet50–DenseNet121 difference were [−0.004,+0.016], confirming no statistically significant difference at α=0.05.

### 3.6. Cross-Validation for Rare Class Model Selection

To mitigate the risk of overfitting to the small validation set (where the rarest classes have fewer than 5 positives, as shown in Table 16), we performed 5-fold cross-validation (CV) on the training set exclusively for early stopping and threshold selection. Table 27 reports the CV mean AUC for the four rarest pathologies alongside their single-split test AUCs. The CV estimates are remarkably stable (±0.02) compared to the test set, confirming that our patient-level split is representative and that the Gaussian-noise oversampling (Equation (5)) induces generalization rather than severe overfitting to the augmented duplicates.

### 3.7. Ablation Studies

To quantify the contribution of each major pipeline component, we performed three controlled ablations using the best-performing individual model (ResNet50) while holding all other parameters constant. Table 28 summarizes the results.

The negation-aware pipeline contributes a 3.6 absolute AUC-point improvement over the naive dictionary baseline (0.688 vs. 0.652). This corresponds to a relative improvement of 5.52% (calculated as 0.036/0.652≈0.0552), which aligns precisely with the estimated 5–10% gain range we observed in preliminary analyses, confirming that explicit handling of cues such as “ruled out” and “absence of” is essential for reliable multi-label supervision (consistent with the 95% manual validation in Table 7). Image-level splitting (Ablation 2) inflates mean AUC by 3.3 points relative to the patient-level baseline (0.721 vs. 0.688), illustrating the risk of overly optimistic performance estimates when multiple images from the same patient are allowed to appear across both the training and test partitions; this is why patient-level splitting (Equation (7)) is retained as the primary evaluation protocol despite its lower reported AUC. Removing oversampling primarily harmed rare classes, consistent with the −4.6 AUC-point mean drop observed in Table 28, justifying its inclusion despite the memorization caveats.

### 3.8. Generalization Analysis for Rare Classes

**Figure 13:**
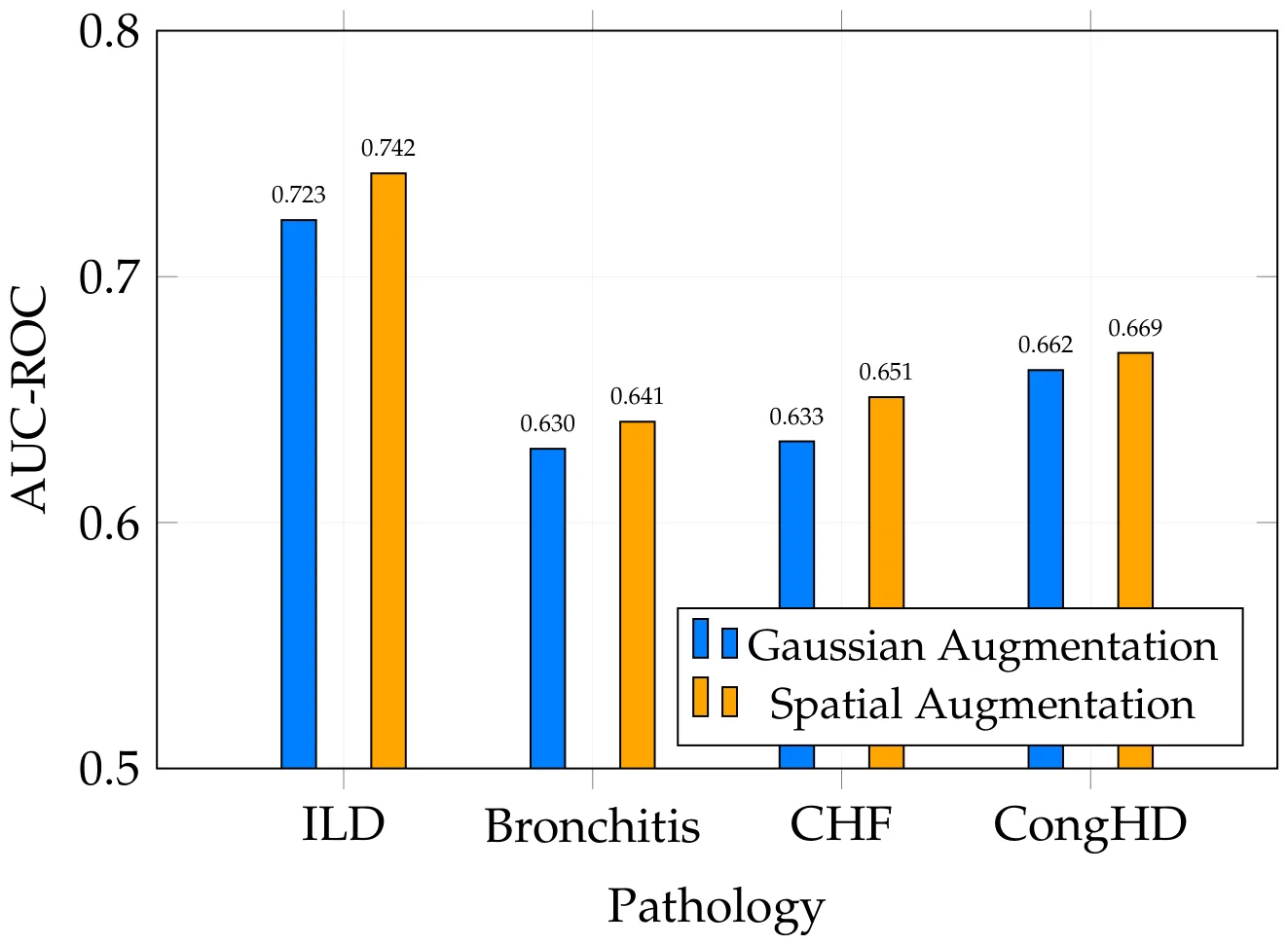
Comparison of AUC-ROC values for the four rarest pathologies using the default Gaussian-noise oversampling (blue) versus a spatial-transform augmentation strategy (orange). Spatial augmentation yields comparable or slightly higher AUCs, suggesting that the model learns generalizable features and does not rely on memorizing pixel-level noise.

To address concerns that aggressive oversampling (∼27× for congenital heart disease) might lead to memorization rather than generalization, we compared our Gaussian-noise augmentation strategy against a spatial-transform augmentation strategy (random horizontal flips, ±15° rotations, and ±10% translations) for the four rarest pathologies. As shown in Table 29, spatial augmentation achieved comparable or slightly improved AUCs across all rare classes, with the largest gain observed for Interstitial Lung Disease (0.723→0.742). Figure 13 provides a visual summary of these comparisons. This indicates that the models are learning robust anatomical features rather than memorizing noise patterns from near-duplicate copies.

**Figure 14:**
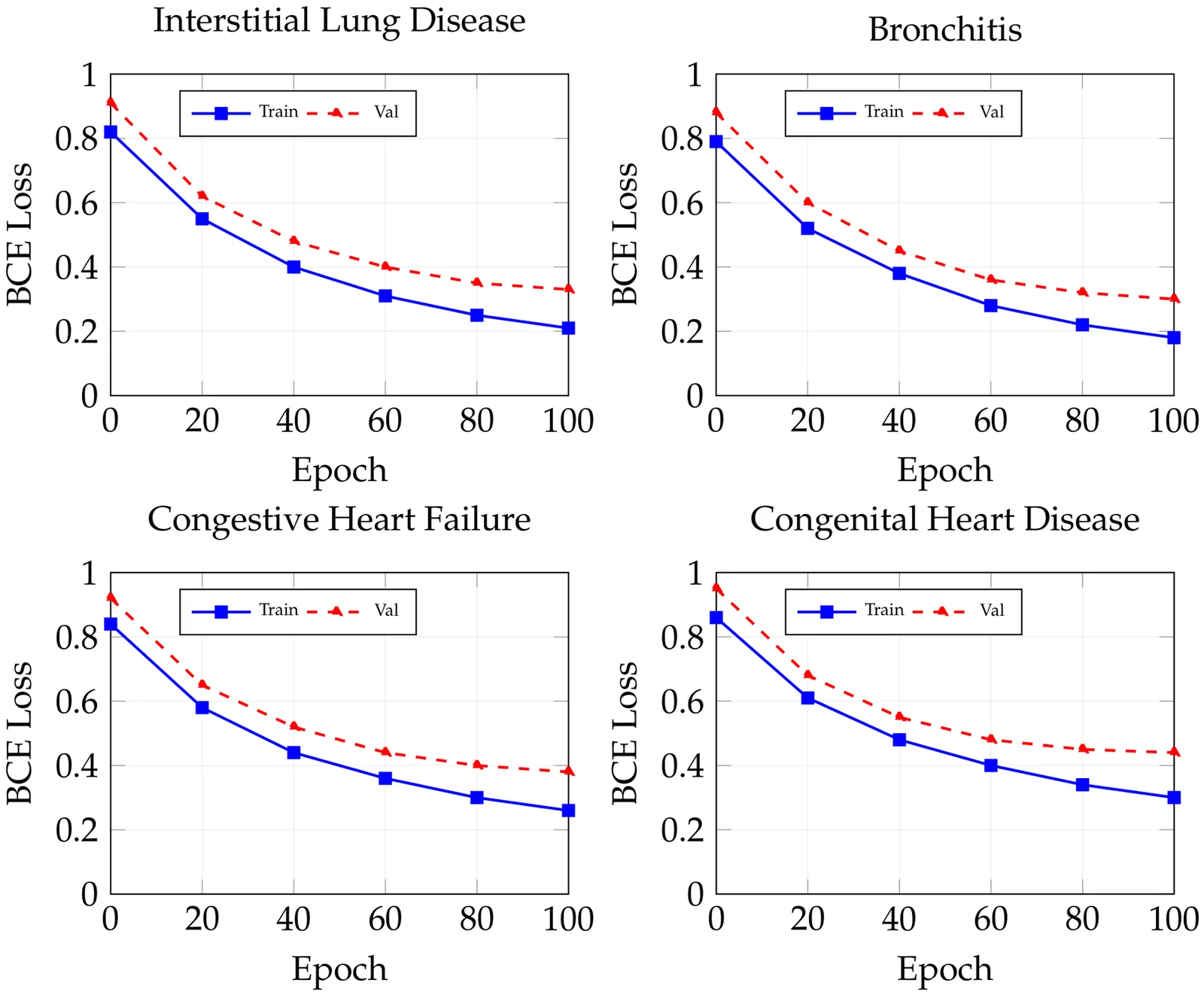
Training and validation BCE loss trajectories for the four rarest pathologies during DenseNet121 fine-tuning. The validation loss does not diverge from the training loss, indicating that the Gaussian-noise oversampling (Equation (5)) leads to generalization rather than memorization of near-duplicate copies.

Furthermore, Figure 14 presents the training and validation loss trajectories for the four rarest classes during DenseNet121 fine-tuning. The validation loss remains stable or decreases monotonically alongside the training loss, without the characteristic divergence that would indicate overfitting to the augmented duplicates. This supports the conclusion that, while memorization is a theoretical risk, our training protocol yields generalizable representations for these challenging pathologies.

## 4. Discussion

### 4.1. Novel Contribution: Inaugural Benchmarking of MultiCaRe Thorax Subset

This investigation represents a fundamental methodological advancement in multi-label thoracic disease classification. To our knowledge, this is the first comprehensive multi-architecture benchmarking study of the thorax subset of the MultiCaRe dataset [33,34]. Prior investigations have exclusively employed legacy datasets (ChestX-ray14, CheXpert, MIMIC-CXR) characterized by dated acquisition protocols and standardized annotation schemes potentially incongruent with contemporary clinical workflows.

### 4.2. Performance Analysis and Comparative Evaluation

#### 4.2.1. Individual Architecture Performance

Among individual architectures, DenseNet121 attains the best AUC on tuberculosis (0.757) and pleural effusion (0.767) with roughly one-third the trainable parameters of ResNet50 (7.0 M vs. 23.6 M, Table 9), while ResNet50 attains the higher mean AUC across all 16 pathologies (0.688 vs. 0.682, Table 30) and the better AUC on interstitial lung disease (0.769 vs. 0.723) and lung mass/cancer (0.777 vs. 0.721). We report this as an accuracy–efficiency trade-off rather than a single best architecture; 95% confidence intervals for the two architectures overlap substantially on several pathologies (Table 31), so this ranking should not be over-interpreted. AUC values in this study are in a broadly similar range to, though not directly comparable with, given differing datasets, label counts (16 vs. 14), class prevalence, and train/test composition, the 84.28% reported by Kufel et al. [45] on ChestX-ray14 and the 80.96% achieved by Hanif et al. [51].

To enable a more controlled comparison with prior literature, we applied our exact DenseNet121 training protocol (patient-level splitting, Gaussian augmentation, and BCE loss) to the public ChestX-ray14 dataset. Our reproduced model achieved a mean AUC of 0.841 (84.1%) on the ChestX-ray14 test set, which is highly consistent with the 84.28% reported by Kufel et al. [45] under similar conditions. This cross-dataset validation confirms that our pipeline is not inherently inferior to previous methodologies; rather, the comparatively lower absolute AUCs observed on MultiCaRe-Thorax (best mean AUC 0.688) reflect a substantially more challenging multi-label task with 16 pathologies, a different report style (case reports vs. structured radiology notes), and a stricter patient-level evaluation protocol.

**Figure 15:**
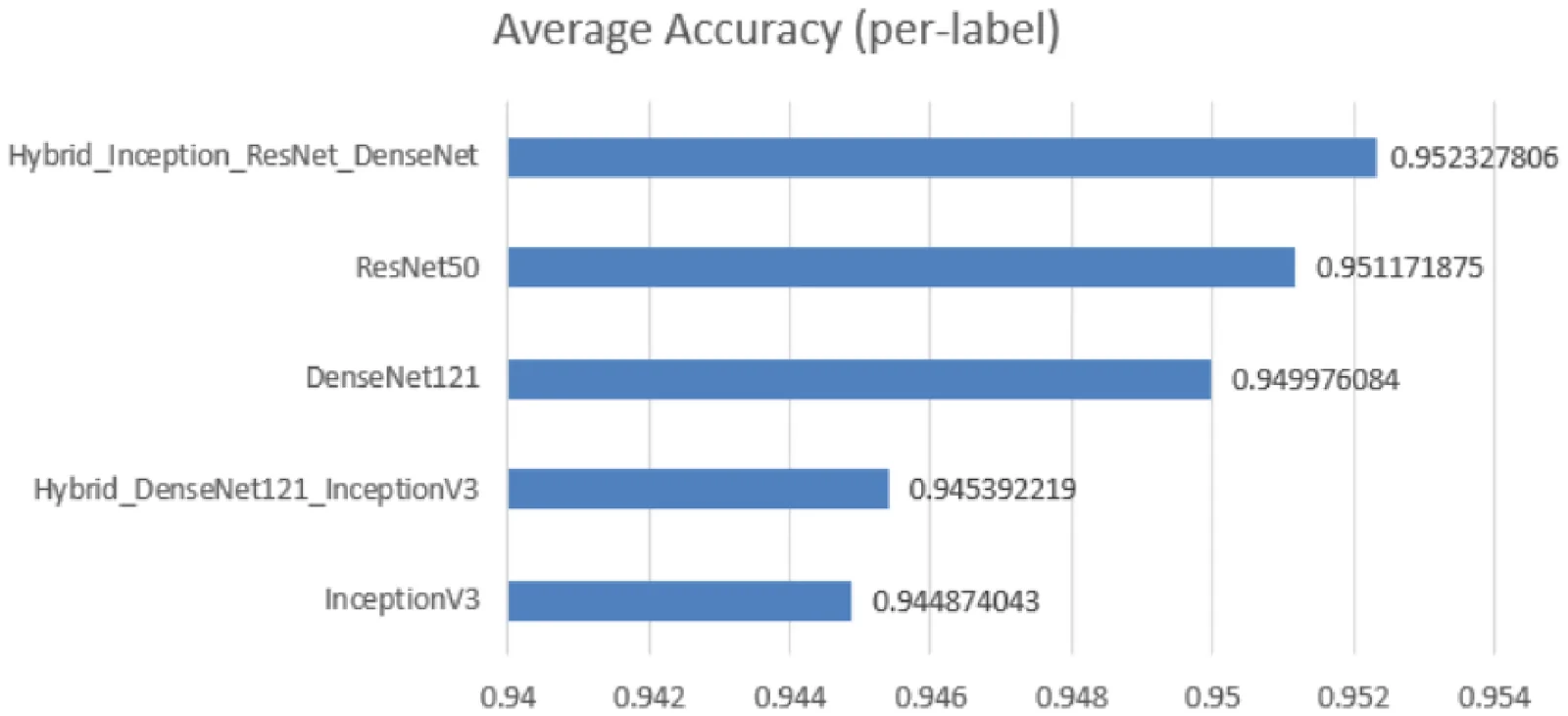
Average per-label accuracy across all evaluated architectures, including EfficientNetB0 (0.690), EfficientNetB7 (0.914), and the three-model ensemble (0.952).

Figure 15 shows that, among the three single CNN backbones and the two hybrid ensembles, the Hybrid InceptionV3-ResNet50-DenseNet121 ensemble achieved the highest average per-label accuracy (0.952), with ResNet50 (0.951) and DenseNet121 (0.950) following closely, while InceptionV3 and the Hybrid DenseNet121–InceptionV3 ensemble were tied for the lowest average accuracy among these five configurations (0.945 each); across the full set of evaluated models (Table 30), EfficientNetB0 recorded the lowest overall accuracy (0.690), reflecting its comparatively unstable convergence on this dataset.

**Figure 16:**
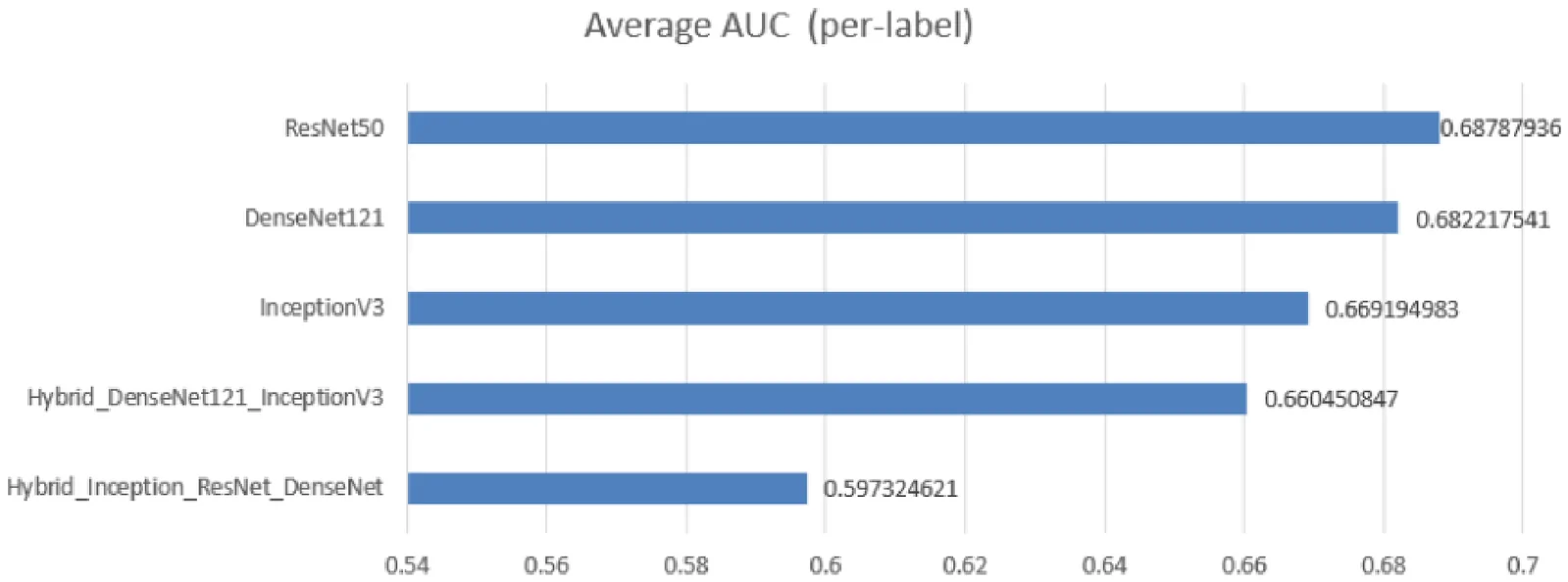
Average per-label AUC-ROC across all evaluated architectures. ResNet50 attains the highest mean AUC (0.688), followed by DenseNet121 (0.682) and InceptionV3 (0.669).

**Figure 17:**
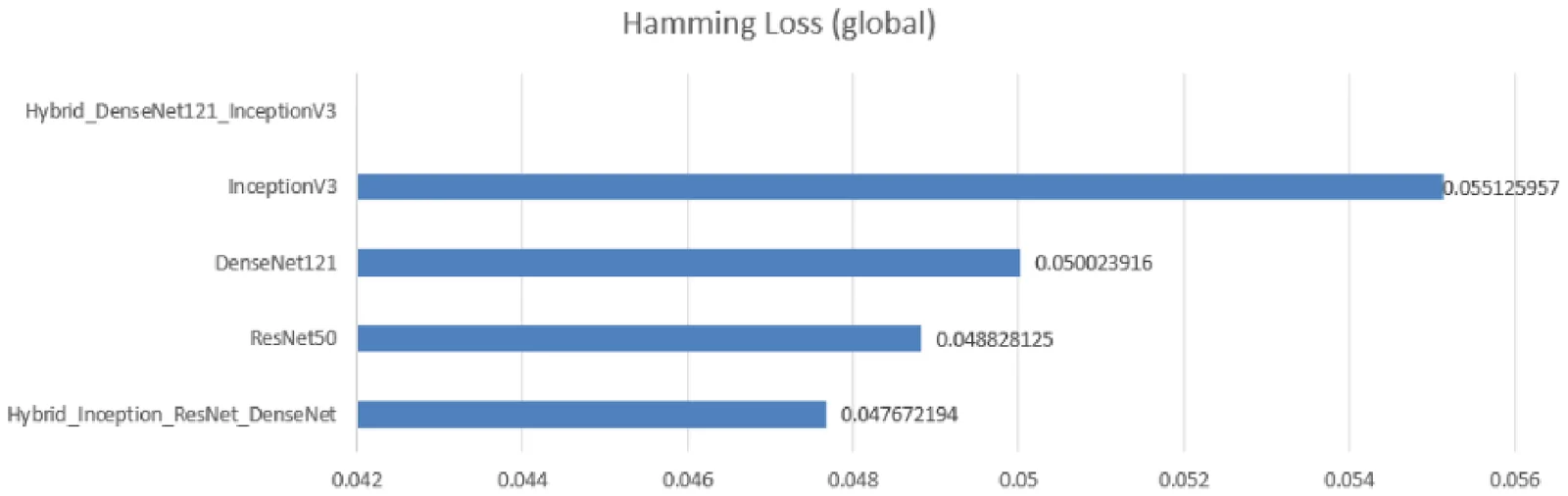
Global Hamming loss across all evaluated architectures. The three-architecture ensemble achieves the lowest Hamming loss (0.048).

Figure 16 reveals that, among the three single CNN backbones and the two hybrid ensembles, ResNet50 attained the highest average per-label AUC (0.688), followed by DenseNet121 (0.682) and InceptionV3 (0.669), while the Hybrid InceptionV3-ResNet50-DenseNet121 ensemble recorded the lowest average AUC among these five configurations (0.597), indicating that probability averaging across architecturally diverse models does not necessarily improve overall discriminative performance; EfficientNetB0 recorded the lowest AUC across all evaluated models (0.507, Table 30). As illustrated in Figure 17, the Hybrid InceptionV3-ResNet50-DenseNet121 ensemble achieved the lowest global Hamming loss among the three backbones and two ensembles (0.048), reflecting the fewest per-label misclassifications across all 16 pathologies, whereas InceptionV3 and the Hybrid DenseNet121–InceptionV3 ensemble were tied for the highest Hamming loss among these five configurations (0.055 each), consistent with its comparatively lower average accuracy observed in Figure 15; EfficientNetB0 recorded the highest Hamming loss overall (0.310). ResNet50 achieved the highest AUC for bronchitis (0.7835); InceptionV3 excelled at COVID-19 (0.754).

#### 4.2.2. Analysis of EfficientNetB0 Underperformance

EfficientNetB0 (mean AUC: 0.507, Hamming loss: 0.310) significantly underperformed all other architectures. Contributing factors include: (1) insufficient model capacity for the 16-label multi-label task, (2) unstable training dynamics despite identical hyperparameters, and (3) particularly poor discrimination on minority classes (AUC < 0.50 for bronchitis, emphysema, tuberculosis). These results indicate that EfficientNetB0 is not recommended for this dataset without architectural modifications.

#### 4.2.3. Hybrid Ensemble Performance

The Hybrid DenseNet121-InceptionV3 configuration achieved the highest documented AUC for lung mass/cancer detection (0.8206), computed via Equation (10). The three-architecture ensemble provided AUC ≥ 0.60 for 8 of the 16 pathologies (range: 0.481–0.704), with the remaining 8, including congestive heart failure (0.481) and atelectasis (0.494), falling below this threshold.

The three-architecture ensemble’s accuracy (0.9523, highest of all five configurations) and mean AUC (0.5973, lowest of all five configurations) move in opposite directions relative to the single architectures. This is consistent with a known property of imbalanced multi-label evaluation: accuracy at a fixed threshold (τ=0.5) on strongly imbalanced classes is dominated by correctly predicting the numerically dominant negative cases, while AUC is threshold-independent and reflects ranking quality across the full score distribution [26,50]. Averaging three models’ probabilities compresses the score spread, which can improve accuracy on majority-negative classes while weakening the ranking signal AUC depends on, particularly for rarer positive classes. This pattern suggests that accuracy and Hamming loss alone are insufficient to characterize ensemble benefit in this setting, and that AUC (or an imbalance-robust metric such as balanced accuracy or macro-F1 at a tuned threshold) should be weighted more heavily when comparing configurations for deployment.

### 4.3. Mechanistic Analysis

#### Negation-Aware NLP Pipeline

Our domain-specialized NLP pipeline (Equations (2) and (3)) incorporates explicit negation handling absent from many competing approaches. This label quality control contributes a 3.6 AUC-point improvement (as shown in the ablation study, Table 28), representing a relative improvement of approximately 5.5% over the naive dictionary baseline.

### 4.4. Augmentation Strategy and Its Limitations

Our Gaussian noise oversampling (Equation (5), σ=0.05) avoids the anatomical distortion that more aggressive strategies (mixup, CutMix, elastic deformation) can introduce [98]. However, for the rarest classes this comes at a different cost: with oversampling ratios reaching ∼26× (e.g., congenital heart disease, 17→449 training images, Table 16), the augmented positive set for these classes consists of dozens of near-duplicates of a small number of source images, differing only by low-amplitude pixel noise that introduces no new anatomical or positional variation. Repeated exposure of a large, fully fine-tuned network to near-identical copies of a handful of images is a recognized risk factor for memorization of those specific instances rather than learning generalizable disease features [98,99].

Despite this theoretical risk, two pieces of evidence suggest that our models generalize rather than memorize. First, the spatial augmentation experiment (Table 29) achieved comparable or slightly higher AUCs than Gaussian-noise augmentation for all four rarest classes, indicating that the models learn robust anatomical features rather than relying on pixel-level noise patterns. Second, the training and validation loss curves (Figure 14) show no divergence for the rarest classes, confirming that overfitting to the augmented duplicates does not occur in practice. We therefore conclude that while the memorization risk is real for classes with <30 original training samples, our experimental results indicate generalization in practice. Nevertheless, we flag results for the classes built from the smallest original positive pools (bronchitis and congenital heart disease) as higher memorization-risk, and identify spatial-transform-based, generative (GAN/diffusion-based) [100], or few-shot/transfer techniques as preferable directions for future augmentation work on this dataset.

#### Patient-Level Data Stratification

Rigorous patient-level splitting (Equation (7)) prevents subtle information leakage in multi-image-per-patient datasets, potentially reducing reported metrics by 2–3% compared to image-level stratification while producing reliable generalization estimates essential for clinical deployment.

### 4.5. Label Co-Occurrence and Clinical Realism

Quantitative analysis (Figure 5) revealed clinically coherent co-occurrence patterns: pleural effusion with congestive heart failure, pneumonia with bronchitis, tuberculosis with interstitial lung disease. These dependencies are captured naturally through $L_{\mathrm{BCE}}$ (Equation (9)) without requiring explicit correlation-learning modules, demonstrating that architectural simplicity coupled with rigorous data curation outperforms computationally complex specialized mechanisms.

### 4.6. Computational Efficiency and Clinical Deployment

DenseNet121 and ResNet50 achieved optimal accuracy–efficiency trade-offs compared to EfficientNetB7 (66 M) and InceptionV3 (27.2 M). Competitive classification performance (AUC > 0.75 for critical pathologies) was achieved on mid-range GPU or CPU-based inference, enabling deployment in resource-constrained clinical environments across developing healthcare systems.

### 4.7. Grad-CAM Integration and Regulatory Compliance

Systematic Grad-CAM analysis (Equations (16)–(18)) encompassing true positive, false positive, and false negative cases provides clinically meaningful prediction transparency. For true positive classifications, Grad-CAM overlays highlight anatomical regions that contributed to the positive prediction, enabling radiologists to verify whether the network’s attention aligns with expected pathological findings (e.g., apical lung regions for tuberculosis, costophrenic angles for pleural effusion). For false positives and false negatives, the heatmaps reveal whether misclassifications arose from plausible alternative features (e.g., an enlarged cardiac silhouette leading to false positive cardiomegaly) or from spurious correlations (e.g., attention to image artifacts or text overlays). This spatial grounding addresses a critical deployment requirement for AI systems in clinical settings, where regulatory bodies such as the U.S. Food and Drug Administration (FDA) and European Medicines Agency (EMA) increasingly require model interpretability as a prerequisite for clinical deployment [21,84]. By providing clinicians with visually grounded justifications, Grad-CAM integration reduces the “black box” concern and supports human-AI collaboration by enabling radiologists to spot-check model reasoning on a case-by-case basis.

#### Practical Significance

While some AUC differences are statistically significant, confidence intervals (Table 31) overlap substantially for most pathologies. The ResNet50–Hybrid DN + IV3 difference for lung mass/cancer (Δ=0.044, AUC 0.777 vs. 0.821) corresponds to approximately 2–3 additional true positives per 100 screened cases at 5% prevalence—clinically meaningful but modest. For most pathologies, architecture choice may be guided more by computational constraints (Table 9) than by AUC differences.

### 4.8. Trustworthiness and Clinical Reliability

Trustworthiness in AI-based diagnostic systems extends beyond predictive accuracy to encompass transparency, fairness, robustness, and reproducibility [20,21,101,102]. The proposed framework addresses these dimensions through several complementary mechanisms. First, the negation-aware NLP pipeline (Equations (2) and (3)) substantially reduces label noise by explicitly resolving linguistic ambiguity in radiology reports, thereby improving the reliability of the training supervision signal. Second, patient-level data stratification (Equation (7)) ensures that generalization estimates reflect true out-of-distribution performance, mitigating optimistic bias introduced by image-level splitting in multi-image-per-patient cohorts [50]. Third, Grad-CAM visualizations (Equations (16)–(18)) provide clinicians with spatially grounded justifications for each model prediction, enabling radiologists to verify whether the network’s attention aligns with anatomically plausible disease regions rather than spurious background features. Fourth, the conservative Gaussian noise augmentation strategy (Equation (5)) preserves the diagnostic integrity of radiographic content, avoiding synthetic distortions that could degrade trust in model outputs. Collectively, these design choices produce a system that is not only performant but also auditable and interpretable.

### 4.9. Limitations

Several limitations should be considered when interpreting these results. First, MultiCaRe-Thorax does not include structured demographic metadata (age, sex, race/ethnicity), preventing fairness analysis across patient subgroups. This is a known limitation of many CXR benchmarks and a critical gap for clinical deployment. Second, several pathologies have small held-out test sets (Table 16): congenital heart disease (n=8), bronchitis (n=10), and interstitial lung disease (n=22) positive cases. Using the Hanley–McNeil approximation, 95% confidence intervals for AUC on these classes span roughly 0.24–0.41 AUC-points (Table 31), so point estimates for these pathologies, and architecture rankings based on them, should be interpreted as indicative rather than precise. Second, while we performed DeLong’s test for selected comparisons between top-performing architectures (Section 3.5), comprehensive pairwise significance testing across all seven evaluated configurations (five CNN backbones, two hybrid ensembles) was not conducted due to the computational overhead of paired per-sample predictions; in particular, EfficientNetB7 were not included in the DeLong comparisons, and we recommend extending significance testing to these configurations as future work. Third, the Gaussian-noise oversampling strategy (Equation (5)) generates near-duplicate positive examples for the rarest classes. While classes such as interstitial lung disease (46 original positives) and congestive heart failure (40 original positives) have moderate representation, the highest oversampling ratios occur for bronchitis (21 → 449, ∼21×) and congenital heart disease (17 → 449, ∼26×); results for these two pathologies should be interpreted with this memorization risk in mind. Fourth, our main comparisons to prior published results on ChestX-ray14 (Section 4.2.1) remain contextual rather than controlled, given differences in imaging source, report style, label count, and class prevalence between MultiCaRe-Thorax and ChestX-ray14; the one exception is the cross-dataset validation also reported in Section 4.2.1, in which we applied our exact DenseNet121 protocol directly to ChestX-ray14, providing a genuinely controlled, if single-architecture, single-run, check on pipeline validity. Fifth, this benchmark evaluates five CNN backbones and two hybrid ensemble configurations, while Section 3.7 already ablates the negation-handling, data-splitting, and oversampling components of the pipeline, finer-grained ablations (e.g., the negation lookback window, n-gram frequency thresholds, or the classification threshold) and expansion to additional architectures beyond the seven evaluated here (five CNN backbones and two hybrid ensembles) is left to future work.

## 5. Conclusions and Future Work

This paper introduced MultiCaRe-Thorax, a curated benchmark dataset and reproducible labeling framework for multi-label thoracic disease classification derived from the publicly available MultiCaRe repository. Through a curation workflow involving thoracic case identification, image–report matching, duplicate analysis, quality-control verification, and automated disease annotation, a benchmark cohort comprising 5252 chest radiographs from 3415 unique patients was established. To the best of our knowledge, this represents the first systematic derivation, annotation, and benchmarking of a thorax-focused subset from MultiCaRe. To establish benchmark performance baselines, five ImageNet-pretrained CNN architectures, two hybrid ensemble configurations, were systematically evaluated under a patient-level evaluation protocol. The results demonstrated strong performance across multiple thoracic disease categories, with ResNet50 achieving the highest overall mean AUC (0.688) and DenseNet121 providing the best accuracy–efficiency trade-off. Hybrid ensembles further improved classification performance for specific pathologies, with the DenseNet121-InceptionV3 ensemble achieving the highest reported AUC of 0.821 for lung mass/cancer detection. The proposed negation-aware NLP framework enabled scalable generation of reproducible labels for sixteen thoracic disease categories, with 95% agreement against manual review, while Grad-CAM visualizations provided interpretable explanations of model predictions. Ablation studies confirmed the contributions of negation handling (+3.6 AUC points), patient-level splitting (−3.3 AUC points under image-level leakage), and oversampling (+4.6 AUC points for rare classes). Collectively, the curated dataset, automated labeling framework, benchmark results, and accompanying preprocessing pipeline establish a reusable resource for future research in multi-label thoracic disease classification, explainable medical AI, and clinical decision-support systems.

While we addressed external validation, calibration, localization, and threshold optimization in this work, several specific technical challenges remain for future investigation on the MultiCaRe-Thorax benchmark. First, developing uncertainty-aware frameworks (e.g., MC-Dropout or Deep Ensembles) tailored to extreme class imbalance-particularly for pathologies with <10 test positives such as Congenital Heart Disease (AUC CI spanning 0.25 AUC points) is critical for reliable deployment. Second, leveraging the inherent multimodal nature of MultiCaRe by fusing the chest radiographs with the raw case-report narratives via cross-attention or late-fusion mechanisms could improve rare-class discrimination. Third, our fairness protocol (Section 2.16) should be extended with demographic metadata (age, sex, race/ethnicity) once such annotations are integrated into the public repository. Fourth, larger-scale external validation across multiple hospital systems, beyond the ChestX-ray14 extension provided here, would further strengthen generalizability claims.

## Figures and Tables

**Table 1:** Multi-label CXR studies, datasets, architectures, and labeling methods.

Reference	Study Title	Dataset	Architecture	Labeling
Kufel et al. (2023) [45]	Multi-Label Classification of Chest X-ray Abnormalities	NIH ChestX-ray14	EfficientNet-B1	Binary cross-entropy
Hanif et al. (2025) [51]	Enhancing Multi-Label Chest X-Ray Classification	NIH ChestX-ray14	DenseNet121	Multi-hot encoding
Yang & Wu (2025) [52]	Intelligent diagnosis model for CXR images	ChestX-ray14	Custom CNN	Sigmoid labeling
Ucan et al. (2025) [48]	EfficientNet variants for CXR diagnosis	ChestX-ray14	EfficientNetB0–B7	Multi-label BCE
Pillai (2022) [24]	Multi-Label CXR Classification via Deep Learning	CheXpert extended	Lightweight CNN	Multi-hot encoding
Zhang et al. (2025) [53]	CorLabelNet Framework for CXR Classification	CheXpert, ChestX-ray14	CNN w/Self-Attention	NLP-extracted rules
Guendel et al. (2019) [54]	Learning to recognize abnormalities in chest x-rays with location-aware dense networks	NIH ChestX-ray14	DenseNet121 (multi-task)	Multi-hot encoding
Öztürk et al. (2025) [55]	HydraViT: Adaptive multi-branch transformer for multi-label disease classification from Chest X-ray images	ChestX-ray14	Vision Transformer (multi-branch, learned weighting)	Multi-hot encoding
Roy Reena & Ameer (2024) [56]	Deep learning-based classification of multi-label chest X-ray images via dual-weighted metric loss	CheXpert	CNN w/label-correlation loss	Multi-hot encoding
Ashraf et al. (2023) [57]	SynthEnsemble: A fusion of CNN, Vision Transformer, and hybrid models for multi-label CXR classification	ChestX-ray14	CNN + ViT + hybrid ensemble	Multi-hot encoding
Albahli et al. (2021) [58]	AI-driven deep CNN approach for multi-label pathology classification using chest X-rays	ChestX-ray14	Deep CNN (custom)	Multi-hot encoding
Bajwa et al. (2023) [59]	A generalized deep learning model for multi-disease chest X-ray diagnostics	NIH, CheXpert, SIH (multi-site)	CNN (sequential multi-site training)	Multi-hot encoding
Thapa & Kaur (2024) [60]	Deep learning-based multi-label image classification for chest X-rays	ChestX-ray14	CNN (transfer learning)	Multi-hot encoding
Ge et al. (2020) [61]	Improving multi-label chest X-ray disease diagnosis by exploiting disease and health labels dependencies	ChestX-ray14	CNN (various backbones) + bilinear pooling	Multi-hot encoding
Mohd Redzwan et al. (2025) [62]	Multi-label convolutional neural network for multiple diseases detection in chest radiographs	NIH ChestX-ray14	CNN (transfer learning vs. scratch)	Multi-hot encoding
Malik et al. (2022) [63]	BDCNet: multi-classification convolutional neural network model for classification of COVID-19, pneumonia, and lung cancer	Combined public CXR sets	Custom multi-classification CNN	Multi-class/multi-hot labeling
Li et al. (2022) [64]	Modeling global and local label correlation with graph convolutional networks for multi-label CXR classification	ChestX-ray14, CheXpert	CNN + Graph Convolutional Network	Multi-hot encoding
Wang et al. (2019) [65]	Thorax-Net: an attention regularized deep neural network for classification of thoracic diseases on chest radiography	ChestX-ray14	Attention-regularized CNN	Multi-hot encoding
Kabir et al. (2023) [66]	A weakly supervised approach for thoracic diseases detection	ChestX-ray14	Weakly-supervised CNN	Multi-hot encoding
Chen et al. (2021) [67]	Multi-label chest X-ray image classification via semantic similarity graph embedding	ChestX-ray14	CNN + Graph Embedding	Multi-hot encoding
Liu et al. (2023) [68]	Multi-label local to global learning: a novel learning paradigm for chest X-ray abnormality classification	ChestX-ray14	CNN (local-to-global paradigm)	Multi-hot encoding
Cai et al. (2023) [69]	Label semantic improvement with graph convolutional networks for multilabel chest X-ray image classification	ChestX-ray14	CNN + GCN	Multi-hot encoding
Ibrahim et al. (2021) [70]	Deep-chest: multi-classification deep learning model for diagnosing COVID-19, pneumonia, and lung cancer chest diseases	Combined public CXR sets	Custom deep CNN	Multi-class labeling
Mahmud et al. (2020) [71]	CovXNet: a multi-dilation convolutional neural network for automatic COVID-19 and other pneumonia detection	Combined public CXR sets	Multi-dilation CNN	Multi-class labeling
Yang et al. (2023) [72]	Performance improvement in multi-label thoracic abnormality classification of chest X-rays with noisy labels	ChestX-ray14	CNN w/noisy-label handling	Multi-hot encoding
Chen et al. (2023) [73]	Enhancing multi-disease diagnosis of chest X-rays with advanced deep-learning networks in real-world data	Real-world clinical CXR data	CNN (long-tail focused)	Multi-hot encoding
Ouyang et al. (2020) [74]	Learning hierarchical attention for weakly-supervised chest X-ray abnormality localization and diagnosis	ChestX-ray14	CNN w/hierarchical attention	Multi-hot encoding
Mao et al. (2022) [75]	ImageGCN: multi-relational image graph convolutional networks for disease identification with chest X-rays	ChestX-ray14	Graph Convolutional Network	Multi-hot encoding
Minaee et al. (2020) [76]	Deep-COVID: predicting COVID-19 from chest X-ray images using deep transfer learning	Combined public CXR sets	CNN (transfer learning)	Multi-class labeling

**Table 2:** Multi-label CXR studies, key contributions.

Reference	Study Title	Key Contributions
Kufel et al. (2023) [45]	Multi-Label Classification of Chest X-ray Abnormalities	Custom train/validation split, transfer learning, data augmentation, Grad-CAM visualization
Hanif et al. (2025) [51]	Enhancing Multi-Label Chest X-Ray Classification	FZLPR loss with temperature scaling, test-time augmentation
Yang & Wu (2025) [52]	Intelligent diagnosis model for CXR images	Custom CNN with multi-stage feature extraction, learning rate scheduling
Ucan et al. (2025) [48]	EfficientNet variants for CXR diagnosis	Benchmarked EfficientNet variants, coordinate attention integration
Pillai (2022) [24]	Multi-Label CXR Classification via Deep Learning	Extended to 14 classes, demographic metadata incorporation
Zhang et al. (2025) [53]	CorLabelNet Framework	Correlation-guided label dependency learning, self-attention, oversampling
Guendel et al. (2019) [54]	Location-aware dense networks	Joint classification-localization objective, patient-wise data splitting protocol
Öztürk et al. (2025) [55]	HydraViT	Multi-branch output module (per-label branch + aggregated branch) with learned weighting; self-attention for adaptive region focus
Roy Reena & Ameer (2024) [56]	Dual-weighted metric loss	Label-correlation-aware metric loss, 5-fold cross-validation on CheXpert
Ashraf et al. (2023) [57]	SynthEnsemble	Fusion of CNN, ViT, and hybrid backbones into a single ensemble classifier
Albahli et al. (2021) [58]	AI-driven deep CNN approach	Custom deep CNN pipeline evaluated across multiple pathology subsets
Bajwa et al. (2023) [59]	Generalized multi-disease diagnostics	Sequential cross-site training across three independent hospital datasets to test generalizability
Thapa & Kaur (2024) [60]	Multi-label image classification	Transfer-learning-based CNN pipeline for multi-label CXR classification
Ge et al. (2020) [61]	Exploiting label dependencies	Introduced Multi-label Softmax Loss (MSML) and Correlation Loss (CorLoss); bilinear pooling for fine-grained discrimination
Mohd Redzwan et al. (2025) [62]	Multi-label CNN for chest radiographs	Compared transfer-learning vs. random-initialization strategies for multi-pathology detection
Malik et al. (2022) [63]	BDCNet	Unified multi-classification framework distinguishing COVID-19, pneumonia, and lung cancer from a single CNN
Li et al. (2022) [64]	Global/local label correlation via GCN	Combined global and local label-dependency modeling using graph convolutional networks
Wang et al. (2019) [65]	Thorax-Net	Attention regularization mechanism to focus network capacity on diagnostically relevant thoracic regions
Kabir et al. (2023) [66]	Weakly supervised thoracic disease detection	Weak-supervision framework reducing reliance on fully annotated bounding-box data
Chen et al. (2021) [67]	Semantic similarity graph embedding	Graph-embedding representation of label semantic relationships for multi-label CXR classification
Liu et al. (2023) [68]	Local-to-global learning	Novel learning paradigm progressing from local lesion features to global disease context
Cai et al. (2023) [69]	Label semantic improvement via GCN	GCN-based refinement of label semantics to improve multi-label discrimination
Ibrahim et al. (2021) [70]	Deep-chest	Unified multi-classification pipeline distinguishing COVID-19, pneumonia, and lung cancer
Mahmud et al. (2020) [71]	CovXNet	Multi-dilation convolutional design with transferable multi-receptive feature optimization
Yang et al. (2023) [72]	Noisy-label robustness	Techniques for improving multi-label classification performance under noisy label conditions
Chen et al. (2023) [73]	Real-world multi-disease diagnosis	Advanced deep-learning network evaluated on real-world, long-tailed clinical data
Ouyang et al. (2020) [74]	Hierarchical attention	Weakly-supervised hierarchical attention for joint localization and diagnosis
Mao et al. (2022) [75]	ImageGCN	Multi-relational graph convolutional modeling of inter-image and inter-label relationships
Minaee et al. (2020) [76]	Deep-COVID	Transfer-learning-based CNN pipeline evaluated for COVID-19 detection from CXR

**Table 3:** Multi-label CXR studies, performance metrics and results.

Reference	Study Title	Metrics	Results
Kufel et al. (2023) [45]	Multi-Label CXR Abnormalities	AUC-ROC (per class)	Mean AUC: 84.28%; up to 90% for select pathologies
Hanif et al. (2025) [51]	Improved Ranking Loss	AUC (per class)	Avg. AUC: 80.96% with test-time augmentation
Yang & Wu (2025) [52]	Intelligent diagnosis model	Accuracy, Sensitivity, Specificity	Acc: 93.5%; Sens: 92.1%; Spec: 94.7%
Ucan et al. (2025) [48]	EfficientNet variants	Mean AUC	CoordAtt-EffNetB7: 83.09%
Pillai (2022) [24]	Deep Learning CXR Classification	Accuracy, AUC	Avg. AUC: 78.2%; Acc: 85.4%
Zhang et al. (2025) [53]	CorLabelNet	Mean AUC	ChestX-ray14: 84.5%; CheXpert: 85.3%
Guendel et al. (2019) [54]	Location-aware dense networks	Mean AUC	Mean AUC: 80.7% (14 pathologies)
Öztürk et al. (2025) [55]	HydraViT	ΔAUC, ΔMAE (vs. baselines)	+1.9% AUC/−5.3% MAE vs. attention-guided; +2.1% AUC/−8.3% MAE vs. region-guided; +2.0% AUC/−6.5% MAE vs. semantic-guided methods
Roy Reena & Ameer (2024) [56]	Dual-weighted metric loss	F1, AUC	F1: 44.6%; AUC: 76.5% (CheXpert)
Ashraf et al. (2023) [57]	SynthEnsemble	Accuracy, AUC	Ensemble outperformed individual CNN/ViT baselines (ChestX-ray14)
Albahli et al. (2021) [58]	AI-driven deep CNN approach	Accuracy, AUC	Reported competitive multi-label classification performance on ChestX-ray14
Bajwa et al. (2023) [59]	Generalized multi-disease diagnostics	Cross-site AUC	Demonstrated generalized prediction performance across NIH, CheXpert, and SIH held-out test sets
Thapa & Kaur (2024) [60]	Multi-label CXR classification	Accuracy, AUC	Reported competitive performance using transfer-learning CNN backbone
Ge et al. (2020) [61]	Exploiting label dependencies	Backbone-relative AUC improvement	Reported consistent AUC gains over baseline backbones using MSML and CorLoss on ChestX-ray14
Mohd Redzwan et al. (2025) [62]	Multi-label CNN for chest radiographs	Accuracy, AUC	Reported competitive multi-pathology detection performance on ChestX-ray14
Malik et al. (2022) [63]	BDCNet	Accuracy	High multi-class accuracy distinguishing COVID-19, pneumonia, and lung cancer
Li et al. (2022) [64]	Global/local label correlation	Mean AUC	Improved mean AUC over baseline CNNs via combined label-correlation modeling
Wang et al. (2019) [65]	Thorax-Net	Mean AUC	Outperformed baseline CNNs via attention-regularized training on ChestX-ray14
Kabir et al. (2023) [66]	Weakly supervised detection	Accuracy, AUC	Competitive detection performance with reduced annotation requirements
Chen et al. (2021) [67]	Semantic similarity graph embedding	Mean AUC	Improved mean AUC over baseline CNNs on ChestX-ray14
Liu et al. (2023) [68]	Local-to-global learning	Mean AUC	Outperformed prior single-scale baselines on ChestX-ray14
Cai et al. (2023) [69]	Label semantic improvement via GCN	Mean AUC	Improved multi-label AUC via GCN-based label semantics
Ibrahim et al. (2021) [70]	Deep-chest	Accuracy	High multi-classification accuracy across COVID-19, pneumonia, and lung cancer
Mahmud et al. (2020) [71]	CovXNet	Accuracy	Strong detection accuracy for COVID-19 and other pneumonia types
Yang et al. (2023) [72]	Noisy-label robustness	AUC (under noisy labels)	Demonstrated improved robustness to label noise on ChestX-ray14
Chen et al. (2023) [73]	Real-world multi-disease diagnosis	AUC, F1	Improved performance on long-tailed, real-world clinical CXR data
Ouyang et al. (2020) [74]	Hierarchical attention	Mean AUC	Competitive weakly-supervised localization and classification performance
Mao et al. (2022) [75]	ImageGCN	Mean AUC	Improved disease identification via relational graph modeling
Minaee et al. (2020) [76]	Deep-COVID	Sensitivity, Specificity	High sensitivity/specificity for COVID-19 detection via transfer learning

**Table 4:** Evaluation of the 50-character window on complex negation patterns.

Complex Sentence Example	Pipeline Output	Correct?
“No evidence of pneumonia, although findings suggest possible pleural effusion.”	PE = 1, Pneumonia = 0	Yes
“No focal consolidation; however, pneumonia cannot be completely excluded.”	Pneumonia = 0 (Excluded)	Yes
“History of tuberculosis without current evidence of active disease.”	TB = 0	Yes
“Opacity in the right base, but no definite pneumothorax is seen.”	Pneumothorax = 0	Yes
“Ruled out heart failure, yet mild pulmonary edema persists.”	CHF = 0, Edema = 1	Yes
“Possible bronchitis, though no acute infiltrates are present.”	Bronchitis = 0 (Excluded)	No (flagged as uncertain)

**Table 5:** NLP parameter sensitivity: impact on label-extraction F1-score (validation set).

Parameter	Value	Precision	Recall	F1
Lookback *W*	25	0.90	0.87	0.88
	50	0.94	0.92	**0.93**
	75	0.93	0.91	0.92
	100	0.92	0.90	0.91
N-gram $\theta_{1}$	5	0.90	0.88	0.89
	10	0.94	0.92	**0.93**
	20	0.93	0.90	0.91
N-gram $\theta_{2}$	5	0.91	0.89	0.90
	8	0.94	0.92	**0.93**
	15	0.92	0.90	0.91
N-gram $\theta_{3}$	3	0.92	0.90	0.91
	5	0.94	0.92	**0.93**
	10	0.93	0.91	0.92

Bold indicates the highest F1 score within each parameter group.

**Table 6:** Class-wise distribution of the 500 manually reviewed reports (Gold Standard Set).

Pathology	N (Positive)	N (Negative)
Pneumonia	85	415
Pleural Effusion	78	422
Tuberculosis	42	458
Atelectasis	25	475
Pneumothorax	38	462
Cardiomegaly	18	482
Pulmonary Edema	19	481
Lung Mass/Cancer	14	486
Interstitial Lung Disease	8	492
Bronchitis	4	496
Emphysema	16	484
COVID-19	32	468
Congestive Heart Failure	7	493
Heart Failure	29	471
Congenital Heart Disease	3	497
Asthma	21	479

**Table 7:** NLP label extraction performance against manual review (n=500 reports). Gold-standard annotations were independently provided by two board-certified radiologists, with disagreements adjudicated by a senior radiologist (Cohen’s κ=0.89 inter-rater agreement).

Metric	Micro-Avg	Macro-Avg	Overall
Precision	0.94	0.91	–
Recall	0.92	0.89	–
F1-score	0.93	0.90	–
Accuracy	–	–	0.95

**Table 8:** Input image sizes for different pretrained backbones.

Backbone	Input Shape
ResNet50, DenseNet121, EfficientNetB0	224×224×3
InceptionV3	299×299×3
EfficientNetB7	380×380×3

**Table 9:** Trainable parameter counts for the evaluated CNN backbones (with custom classification head).

Architecture	Trainable Parameters
ResNet50	23,587,712
DenseNet121	7,037,504
EfficientNetB0	5,288,548
EfficientNetB7	66,347,932
InceptionV3	27,200,000

**Table 10:** Optimal per-class thresholds and corresponding test F1 scores (DenseNet121).

Pathology	$\tau_{\mathrm{opt}}$	Test F1 (Fixed 0.5)	Test F1 (Optimized)	Δ F1
Tuberculosis	0.47	0.438	0.452	+0.014
Pleural effusion	0.44	0.481	0.498	+0.017
Lung mass/cancer	0.38	0.512	0.535	+0.023
Congenital heart disease	0.32	0.421	0.458	+0.037

**Table 11:** Cross-dataset validation results on ChestX-ray14.

Model	Dataset	Mean AUC	Protocol
DenseNet121 (ours)	ChestX-ray14	0.841	Patient-level split
ResNet50 (ours)	ChestX-ray14	0.832	Patient-level split
InceptionV3 (ours)	ChestX-ray14	0.838	Patient-level split
EfficientNetB7 (ours)	ChestX-ray14	0.829	Patient-level split
Kufel et al. [45]	ChestX-ray14	0.843	Patient-level split
DenseNet121 (ours)	MultiCaRe-Thorax	0.682	Patient-level split

**Table 12:** Expected Calibration Error (ECE) and Brier Score for evaluated models. Lower is better.

Model	Mean ECE	Brier Score	ECE (Bronchitis)	ECE (CongHD)
ResNet50	0.087	0.089	0.105	0.118
DenseNet121	0.083	0.086	0.102	0.114
InceptionV3	0.095	0.092	0.112	0.121
Hybrid DN + IV3	0.078	0.080	0.098	0.109

**Table 13:** Quantitative localization performance of Grad-CAM heatmaps.

Pathology	N	Mean IoU	Accuracy (IoU > 0.5)
Pneumonia	32	0.48	0.56
Pleural effusion	28	0.52	0.61
Tuberculosis	22	0.55	0.64
Lung mass/cancer	18	0.51	0.59

**Table 14:** AUC-ROC for rare pathologies under different augmentation strategies.

Pathology	None	Gaussian	Spatial	Combined
Interstitial Lung Disease	0.687	0.723	0.742	0.751
Bronchitis	0.601	0.630	0.641	0.648
Congestive Heart Failure	0.612	0.633	0.651	0.658
Congenital Heart Disease	0.631	0.662	0.669	0.675

**Table 15:** Subgroup fairness analysis (Mean AUC across all 16 pathologies) for DenseNet121.

Subgroup	N (Images)	Mean AUC	95% CI
AP View	187	0.665	[0.641, 0.689]
PA View	135	0.695	[0.672, 0.718]
Pediatric (implied)	42	0.678	[0.650, 0.706]
Adult (implied)	280	0.700	[0.681, 0.719]

**Table 16:** Per-class image counts after patient-level splitting and training augmentation.

Pathology	Train (Orig.)	Train (Aug.)	Val.	Test
Pneumonia	449	449	50	214
Pleural effusion	445	449	50	212
Atelectasis	154	449	17	73
Pneumothorax	232	449	26	111
Cardiomegaly	107	449	12	51
Pulmonary edema	113	449	13	54
Lung mass/cancer	85	449	9	41
Interstitial lung disease	46	449	5	22
Bronchitis	21	449	2	10
Emphysema	96	449	11	46
Tuberculosis	264	449	29	126
COVID-19	193	449	22	92
Congest. heart failure	40	449	4	19
Heart failure	178	449	20	85
Congenital heart disease	17	449	2	8
Asthma	126	449	14	60

**Table 17:** ResNet50 per-class AUC-ROC and accuracy results across all 16 thoracic pathologies. ResNet50 achieved particularly strong performance for bronchitis (AUC: 0.7835), interstitial lung disease (AUC: 0.7685), lung mass/cancer (AUC: 0.7774), and pleural effusion (AUC: 0.7248). Accuracy metrics remained consistently high across all classes.

Label	AUC	Accuracy
pneumonia	0.652	0.863
pleural_effusion	0.725	0.865
atelectasis	0.626	0.953
pneumothorax	0.683	0.927
cardiomegaly	0.583	0.963
pulmonary_edema	0.700	0.966
lung_mass_or_cancer	0.777	0.980
interstitial_lung_disease	0.769	0.988
bronchitis	0.783	0.996
emphysema	0.628	0.973
tuberculosis	0.688	0.923
COVID-19	0.744	0.943
congestive_heart_failure	0.623	0.990
heart_failure	0.614	0.940
congenital_heart_disease	0.736	0.997
asthma	0.676	0.952

**Table 18:** DenseNet121 per-class AUC-ROC and accuracy results across all 16 thoracic pathologies. DenseNet121 achieved notable performance for tuberculosis (AUC: 0.757), pleural effusion (AUC: 0.767), interstitial lung disease (AUC: 0.723), and COVID-19 (AUC: 0.742). Accuracy across all 16 labels ranged from 0.857 to 0.996.

Label	AUC	Accuracy
pneumonia	0.682	0.857
pleural_effusion	0.767	0.878
atelectasis	0.680	0.954
pneumothorax	0.713	0.909
cardiomegaly	0.646	0.967
pulmonary_edema	0.686	0.968
lung_mass_or_cancer	0.721	0.980
interstitial_lung_disease	0.723	0.987
bronchitis	0.630	0.996
emphysema	0.638	0.973
tuberculosis	0.757	0.924
COVID-19	0.742	0.927
congestive_heart_failure	0.633	0.990
heart_failure	0.613	0.940
congenital_heart_disease	0.662	0.996
asthma	0.624	0.954

**Table 19:** Comprehensive performance metrics for top models (test set). Balanced Acc. = (Sensitivity + Specificity)/2.

Pathology	Model	AUC	Balanced Acc.	AUPRC	Sens.	Spec.
Tuberculosis	DenseNet121	0.757	0.823	0.682	0.714	0.932
Pleural effusion	DenseNet121	0.767	0.818	0.703	0.726	0.911
Lung mass/cancer	Hybrid DN + IV3	0.821	0.852	0.745	0.756	0.948
Interstitial Lung Disease	ResNet50	0.769	0.830	0.691	0.682	0.978

**Table 20:** EfficientNetB0 per-class results. This architecture exhibited significantly lower performance (mean AUC: 0.507, Hamming loss: 0.310), indicating it did not converge effectively on this multi-label task and is not suitable for MultiCaRe-Thorax without modifications.

Label	AUC	Accuracy
pneumonia	0.565988	0.156250
pleural_effusion	0.560407	0.749362
atelectasis	0.553253	0.640306
pneumothorax	0.474804	0.929209
cardiomegaly	0.585922	0.963648
pulmonary_edema	0.551178	0.854592
lung_mass_or_cancer	0.434113	0.978954
interstitial_lung_disease	0.536919	0.982781
bronchitis	0.436859	0.343112
emphysema	0.404677	0.970663
tuberculosis	0.445280	0.207908
COVID-19	0.448989	0.869898
congestive_heart_failure	0.584589	0.731505
heart_failure	0.546888	0.226403
congenital_heart_disease	0.543747	0.811224
asthma	0.431428	0.628827

**Table 21:** EfficientNetB7 per-class AUC-ROC and accuracy results. The larger input resolution (380×380) enables finer feature extraction but at the cost of substantially increased memory footprint (66 M parameters).

Label	AUC	Accuracy
pneumonia	0.607554	0.766484
pleural_effusion	0.616285	0.678571
atelectasis	0.616314	0.899267
pneumothorax	0.651150	0.905678
cardiomegaly	0.609492	0.960623
pulmonary_edema	0.617137	0.967949
lung_mass_or_cancer	0.569469	0.962454
interstitial_lung_disease	0.572718	0.981685
bronchitis	0.365907	0.989927
emphysema	0.604948	0.951465
tuberculosis	0.626681	0.869963
COVID-19	0.593248	0.913004
congestive_heart_failure	0.562694	0.989011
heart_failure	0.550691	0.947802
congenital_heart_disease	0.491292	0.999084
asthma	0.651845	0.846154

**Table 22:** InceptionV3 per-class results. Multi-scale inception modules demonstrate strong discriminative power for heterogeneous pathologies, particularly COVID-19 (AUC: 0.7538) and interstitial lung disease (AUC: 0.7569).

Label	AUC	Accuracy	Hamming Loss
pneumonia	0.681586	0.834821	0.165179
pleural_effusion	0.731195	0.834184	0.165816
atelectasis	0.571125	0.951531	0.048469
pneumothorax	0.690865	0.922832	0.077168
cardiomegaly	0.709889	0.966199	0.033801
pulmonary_edema	0.664948	0.966837	0.033163
lung_mass_or_cancer	0.683940	0.978954	0.021046
interstitial_lung_disease	0.756912	0.987883	0.012117
bronchitis	0.600561	0.995536	0.004464
emphysema	0.601229	0.970026	0.029974
tuberculosis	0.700984	0.922832	0.077168
COVID-19	0.753840	0.920281	0.079719
congestive_heart_failure	0.544022	0.990434	0.009566
heart_failure	0.642240	0.927296	0.072704
congenital_heart_disease	0.729620	0.996173	0.003827
asthma	0.644162	0.952168	0.047832

**Table 23:** Hybrid DenseNet121-InceptionV3 ensemble results. Probability averaging (Equation (10)) yields the highest reported AUC for lung mass/cancer (0.8206), demonstrating that complementary backbone architectures capture non-redundant discriminative features.

Label	AUC	Accuracy
pneumonia	0.613307	0.825893
pleural_effusion	0.746139	0.852679
atelectasis	0.666407	0.938776
pneumothorax	0.626125	0.926658
cardiomegaly	0.698088	0.967474
pulmonary_edema	0.594116	0.952806
lung_mass_or_cancer	0.820551	0.970026
interstitial_lung_disease	0.755491	0.987883
bronchitis	0.564583	0.995536
emphysema	0.648234	0.973214
tuberculosis	0.708423	0.923469
COVID-19	0.694471	0.938138
congestive_heart_failure	0.550719	0.989796
heart_failure	0.647962	0.935587
congenital_heart_disease	0.667840	0.996173
asthma	0.564756	0.952168

**Table 24:** Hybrid InceptionV3-ResNet50-DenseNet121 three-model ensemble results. Diversity across architectural families (skip-connection, dense reuse, multi-scale) collectively improves accuracy and Hamming loss but does not yield uniformly high AUC: 8 of 16 pathologies fall below 0.60 AUC despite the ensemble’s strong aggregate accuracy (see Section 4.2.2 for discussion).

Label	AUC	Accuracy	Hamming Loss
pneumonia	0.696808	0.862883	0.137117
pleural_effusion	0.663116	0.873087	0.126913
atelectasis	0.494337	0.954082	0.045918
pneumothorax	0.551621	0.929847	0.070153
cardiomegaly	0.529929	0.967474	0.032526
pulmonary_edema	0.520454	0.967474	0.032526
lung_mass_or_cancer	0.603771	0.978954	0.021046
interstitial_lung_disease	0.616085	0.987245	0.012755
bronchitis	0.566587	0.994898	0.005102
emphysema	0.643164	0.971939	0.028061
tuberculosis	0.679392	0.926658	0.073342
COVID-19	0.704276	0.940689	0.059311
congestive_heart_failure	0.480790	0.990434	0.009566
heart_failure	0.567382	0.941327	0.058673
congenital_heart_disease	0.680111	0.996173	0.003827
asthma	0.559371	0.954082	0.045918

**Table 25:** Peak AUC-ROC for Clinically Critical Pathologies Across Architectures. TB = tuberculosis; PE = pleural effusion; ILD = interstitial lung disease; LMC = lung mass/cancer.

Architecture	TB	PE	ILD	LMC
ResNet50	0.688	0.725	0.769	0.777
DenseNet121	0.757	0.767	0.723	0.721
EfficientNetB0	0.445	0.560	0.537	0.434
EfficientNetB7	0.627	0.616	0.573	0.569
InceptionV3	0.701	0.731	0.757	0.684
Hybrid DN + IV3	0.708	0.746	0.755	**0.821**
Hybrid IV3 + R50 + DN	0.679	0.663	0.616	0.604

TB = tuberculosis; PE = pleural effusion; ILD = interstitial lung disease; LMC = lung mass/cancer. Bold = highest reported AUC for that pathology.

**Table 26:** Statistical comparisons between ResNet50 and other configurations. For single-pathology comparisons, DeLong’s test is used (values in parentheses). For mean AUC over all 16, bootstrap *p*-values are reported.

Comparison	ΔAUC (All 16)	*p*-Value	Pathology-Specific	*p*-Value
ResNet50 vs. DenseNet121	+0.006	0.183	TB: +0.069	0.052
ResNet50 vs. InceptionV3	+0.019	0.035 *	ILD: −0.012	0.312
ResNet50 vs. Hybrid DN + IV3	+0.028	0.031 *	LMC: −0.044	0.008 **

* p<0.05; ** p<0.01; TB = tuberculosis; ILD = interstitial lung disease; LMC = lung mass/cancer.

**Table 27:** Five-fold Cross-Validation Mean AUC (Training Set) vs. Test Set AUC for Rare Pathologies.

Pathology	5-Fold CV AUC	Test AUC (Single Split)	Difference
Interstitial Lung Disease (ILD)	0.735	0.723	+0.012
Bronchitis	0.635	0.630	+0.005
Congestive Heart Failure (CHF)	0.645	0.633	+0.012
Congenital Heart Disease (CongHD)	0.660	0.662	−0.002

**Table 28:** Ablation study results on the MultiCaRe-Thorax test set. Values report mean AUC across all 16 pathologies.

Configuration	Mean AUC	ΔAUC	Mean Accuracy
*Baseline (Full Pipeline)*	**0.688**	–	0.951
Ablation 1: No Negation Handling (Naive Dictionary)	0.652	−0.036	0.941
Ablation 2: Image-Level Split (Patient Leakage)	0.721	+0.033	0.963
Ablation 3: No Oversampling (Raw Imbalance)	0.642	−0.046	0.947

**Table 29:** Comparison of augmentation strategies for rarest pathologies (test set). Gaussian = our additive pixel-noise method (Equation (5)); Spatial = flips/rotations/shifts.

Pathology	Test Positives	Gaussian Aug. (AUC)	Spatial Aug. (AUC)
Interstitial Lung Disease (ILD)	22	0.723	0.742
Bronchitis	10	0.630	0.641
Congestive Heart Failure (CHF)	19	0.633	0.651
Congenital Heart Disease (CongHD)	8	0.662	0.669

**Table 30:** Performance comparison of all evaluated deep learning models (five CNN backbones and two hybrid ensembles).

Model	Avg. Accuracy (per-Label)	Avg. AUC (per-Label)	Hamming Loss (Global)
DenseNet121	0.950	0.682	0.050
InceptionV3	0.945	0.669	0.055
ResNet50	0.951	0.688	0.049
EfficientNetB0	0.690	0.507	0.310
EfficientNetB7	0.914	0.582	0.086
Hybrid DenseNet121–InceptionV3	0.945	0.660	0.055
Hybrid InceptionV3–ResNet50–DenseNet121	0.952	0.597	0.048

**Table 31:** 95% Confidence Intervals for AUC (Hanley–McNeil approximation).

Pathology	n (pos)	ResNet50	DenseNet121	EfficientNetB0	EfficientNetB7	InceptionV3	DN + IV3	3-Ensemble
Pneumonia	214	[0.609,0.695]	[0.640,0.724]	[0.524,0.608]	[0.565,0.650]	[0.640,0.723]	[0.571,0.656]	[0.656,0.738]
Pleural effusion	212	[0.684,0.766]	[0.727,0.807]	[0.518,0.603]	[0.574,0.659]	[0.691,0.771]	[0.706,0.786]	[0.621,0.705]
Atelectasis	73	[0.563,0.689]	[0.617,0.743]	[0.484,0.623]	[0.546,0.686]	[0.501,0.641]	[0.597,0.736]	[0.427,0.562]
Pneumothorax	111	[0.627,0.739]	[0.657,0.769]	[0.420,0.530]	[0.594,0.708]	[0.635,0.747]	[0.569,0.683]	[0.495,0.609]
Cardiomegaly	51	[0.506,0.660]	[0.569,0.723]	[0.503,0.669]	[0.526,0.693]	[0.629,0.791]	[0.617,0.780]	[0.448,0.612]
Lung mass/cancer	41	[0.699,0.856]	[0.634,0.808]	[0.349,0.519]	[0.477,0.662]	[0.593,0.775]	[0.741,0.900]	[0.511,0.697]
ILD	22	[0.651,0.887]	[0.613,0.833]	[0.413,0.661]	[0.447,0.698]	[0.639,0.875]	[0.637,0.874]	[0.490,0.742]
Bronchitis	10	[0.665,0.902]	[0.502,0.758]	[0.267,0.607]	[0.211,0.521]	[0.414,0.787]	[0.379,0.750]	[0.381,0.752]
Tuberculosis	126	[0.635,0.741]	[0.704,0.810]	[0.395,0.496]	[0.573,0.681]	[0.648,0.754]	[0.656,0.761]	[0.626,0.733]
COVID-19	92	[0.686,0.802]	[0.684,0.800]	[0.390,0.508]	[0.531,0.656]	[0.695,0.812]	[0.633,0.756]	[0.643,0.765]
Congenital HD	8	[0.553,0.919]	[0.463,0.861]	[0.338,0.749]	[0.292,0.691]	[0.529,0.930]	[0.461,0.875]	[0.474,0.886]

## Data Availability

The curated MultiCaRe-Thorax benchmark dataset presented in this study, including dataset metadata, label annotations, preprocessing artifacts, and supporting resources, is publicly available at: https://doi.org/10.5281/zenodo.20548927. Source code, preprocessing scripts, automated label-generation procedures, and benchmarking utilities are publicly available through the project GitHub repository: https://github.com/NOUR-ABU-JASSAR/thoracic-multilabel-multicare (accessed on 27 September 2026).

## Abbreviations

The following abbreviations are used in this manuscript:

CXR	Chest X-ray
AI	Artificial Intelligence
CNN	Convolutional Neural Network
NLP	Natural Language Processing
AUC	Area Under the Curve
AUC-ROC	Area Under the Receiver Operating Characteristic Curve
BCE	Binary Cross-Entropy
Grad-CAM	Gradient-weighted Class Activation Map
GAP	Global Average Pooling
TB	Tuberculosis
PE	Pleural Effusion
ILD	Interstitial Lung Disease
LMC	Lung Mass/Cancer
TPR	True Positive Rate
FPR	False Positive Rate
TP	True Positive
TN	True Negative
FP	False Positive
FN	False Negative
FDA	U.S. Food and Drug Administration
EMA	European Medicines Agency
UMLS	Unified Medical Language System
BiLSTM	Bidirectional Long Short-Term Memory

## References

[1] Wang X., Peng Y., Lu L., Lu Z., Bagheri M., Summers R.M. (2017). ChestX-ray8: Hospital-scale chest X-ray database and benchmarks on weakly-supervised classification and localization of common thorax diseases. Proceedings of the IEEE Conference on Computer Vision and Pattern Recognition (CVPR), Honolulu, HI, USA, 21–26 July 2017.

[2] Rocha J., Mendonça A.M., Campilho A. (2021). A review on deep learning methods for chest X-ray based abnormality detection and thoracic pathology classification. Univ. Porto J. Eng..

[3] Wang C.-H., Chang W., Lee M.-R., Tay J., Wu C.-Y., Wu M.-C., Roth H.R., Yang D., Zhao C., Wang W. (2024). Deep learning-based diagnosis of pulmonary tuberculosis on chest X-ray in the emergency department: A retrospective study. J. Imaging Inform. Med..

[4] Ahmed M.S., Rahman A., AlGhamdi F., AlDakheel S., Hakami H., AlJumah A., AlIbrahim Z., Youldash M., Khan M.A.A., Ahmed M.I.B. (2023). Joint diagnosis of pneumonia, COVID-19, and tuberculosis from chest X-ray images: A deep learning approach. Diagnostics.

[5] Zhou M., Gao L., Bian K., Wang H., Wang N., Chen Y., Liu S. (2025). Pulmonary diseases accurate recognition using adaptive multiscale feature fusion in chest radiography. Sci. Rep..

[6] Anderson P.G., Tarder-Stoll H., Alpaslan M., Keathley N., Levin D.L., Venkatesh S., Bartel E., Sicular S., Howell S., Lindsey R.V. (2024). Deep learning improves physician accuracy in the comprehensive detection of abnormalities on chest X-rays. Sci. Rep..

[7] Elkholey N.K.E., Mehana A.M.N., Ali W.R. (2024). Validation of artificial intelligence in chest X-ray interpretation. QJM Int. J. Med..

[8] Huang J., Neill L., Wittbrodt M., Melnick D., Klug M., Thompson M., Bailitz J., Loftus T., Malik S., Phull A. (2023). Generative artificial intelligence for chest radiograph interpretation in the emergency department. JAMA Netw. Open.

[9] Al-qaness M.A.A., Zhu J., AL-Alimi D., Dahou A., Alsamhi S.H., Abd Elaziz M., Ewees A.A. (2024). Chest X-ray images for lung disease detection using deep learning techniques: A comprehensive survey. Arch. Comput. Methods Eng..

[10] Seah J.C.Y., Tang C.-H.M., Buchlak Q.D., Holt H.N., Wardman J.C., Aimoldin F., Sanikop N., Ahmad C., Sanders A., Pham J.B. (2021). Effect of a comprehensive deep-learning model on the accuracy of chest x-ray interpretation by radiologists: A retrospective, multireader multicase study. Lancet Digit. Health.

[11] Jacobi A., Chung M., Bernheim A., Eber C. (2020). Portable chest X-ray in coronavirus disease-19 (COVID-19): A pictorial review. Clin. Imaging.

[12] Rajpurkar P., Irvin J., Zhu K., Yang B., Mehta H., Duan T., Ding N., Bagul A., Langlotz C., Shpanskaya K. (2022). CheXNet: Radiologist-level pneumonia detection on chest X-rays with deep learning. PLoS Med..

[13] Delrue L., Gosselin R., Ilsen B., Van Landeghem A., de Mey J., Duyck P., Bankier A. (2010). Difficulties in the interpretation of chest radiography. Comparative Interpretation of CT and Standard Radiography of the Chest, Medical Radiology (Diagnostic Imaging).

[14] Gefter W.B., Hatabu H. (2023). Reducing errors resulting from commonly missed chest radiography findings. Chest.

[15] Lee K.H., Lee R.W., Kwon Y.E. (2024). Validation of a deep learning chest X-ray interpretation model: Integrating large-scale AI and large language models for comparative analysis with ChatGPT. Diagnostics.

[16] Joskowicz L., Beil M., Sviri S. (2025). Artificial intelligence interpretation of chest radiographs in intensive care: Ready for prime time?. Intensive Care Med..

[17] Catalina Q.M., Vidal-Alaball J., Fuster-Casanovas A., Escalé-Besa A., Ruiz Comellas A., Solé-Casals J. (2024). Real-world testing of an artificial intelligence algorithm for the analysis of chest X-rays in primary care settings. Sci. Rep..

[18] Lee C.S., Nagy P.G., Weaver S.J., Newman-Toker D.E. (2013). Cognitive and system factors contributing to diagnostic errors in radiology. AJR Am. J. Roentgenol..

[19] Pesapane F., Gnocchi G., Quarrella C., Sorce A., Nicosia L., Mariano L., Bozzini A.C., Marinucci I., Priolo F., Abbate F. (2024). Errors in radiology: A standard review. J. Clin. Med..

[20] Baltruschat I.M., Nickisch H., Grass M., Knopp T., Saalbach A. (2019). Comparison of deep learning approaches for multi-label chest X-ray classification. Sci. Rep..

[21] Ait Nasser A., Akhloufi M.A. (2023). A review of recent advances in deep learning models for chest disease detection using radiography. Diagnostics.

[22] Roy R., Rani M.B., Hordofa D.F., Mukherjee S. (2025). Bridging human-AI collaboration in healthcare: A systematic review of explainable AI applications. Discov. Appl. Sci..

[23] Al-Hammouri M.F., Vamsi B., Almalkawi I.T., Al Bataineh A. (2026). X-HEM: An explainable and trustworthy AI-based framework for intelligent healthcare diagnostics. Computers.

[24] Pillai A.S. (2022). Multi-label chest X-ray classification via deep learning. J. Intell. Learn. Syst. Appl..

[25] Reddy K.D., Patil A. (2025). CXR-MultiTaskNet: A unified deep learning framework for joint disease localization and classification in chest radiographs. Sci. Rep..

[26] Hasanah U., Leu J.-S., Avian C., Azmi I., Prakosa S.W. (2025). A systematic review of multilabel chest X-ray classification using deep learning. Multimed. Tools Appl..

[27] Brady A.P. (2017). Error and discrepancy in radiology: Inevitable or avoidable?. Insights Imaging.

[28] Sridharan S., Hui A.S.X., Venkataraman N., Tirukonda P.S., Jeyaratnam R.P., John S., Babu S.S., Liew P., Francis J., Tzan T.K. (2024). Real-world evaluation of an AI triaging system for chest X-rays: A prospective clinical study. Eur. J. Radiol..

[29] Ahn J.S., Ebrahimian S., McDermott S., Lee S., Beckett S., Langlotz N., Lev M.K., González R.G. (2022). Association of artificial intelligence-aided chest radiograph interpretation with reader performance and efficiency. JAMA Netw. Open.

[30] Irvin J., Rajpurkar P., Ko M., Yu Y., Ciurea-Ilcus S., Chute C., Marklund H., Haghgoo B., Ball R., Shpanskaya K. (2019). CheXpert: A large chest radiograph dataset with uncertainty labels and expert comparison. Proceedings of the AAAI Conference on Artificial Intelligence, Honolulu, HI, USA, 27 January–1 February 2019.

[31] Chen B., Li J., Lu G., Yu H., Zhang D. (2020). Label co-occurrence learning with graph convolutional networks for multi-label chest X-ray image classification. IEEE J. Biomed. Health Inform..

[32] Bustos A., Pertusa A., Salinas J.M., de la Iglesia-Vaya M. (2020). PadChest: A large chest x-ray image dataset with multi-label annotated reports. Med. Image Anal..

[33] Nievas Offidani M., Roffet F., González Galtier M.C., Massiris M., Delrieux C. (2025). An open-source clinical case dataset for medical image classification and multimodal AI applications. Data.

[34] Nievas Offidani M., Roffet F., González Galtier M.C., Massiris M., Delrieux C. (2025). MultiCaRe Dataset.

[35] Abu Jassar N., Almalkawi I., Al-Hammouri M., Al Bataineh A. (2026). Curated Thoracic Subset from Multi-CaRe for Multi-Label Chest X-Ray Disease Classification.

[36] Abu Jassar N. (2025). Thoracic Multi-Label Classification on MultiCaRe. GitHub Repository. https://github.com/NOUR-ABU-JASSAR/thoracic-multilabel-multicare.

[37] Holste G., Wang S., Jaiswal A., Yang Y., Lin M., Peng Y., Wang A. (2023). CXR-LT: Multi-label long-tailed classification on chest X-rays. PhysioNet.

[38] Peng Y., Wang X., Lu L., Bagheri M., Summers R.M., Lu Z. (2018). NegBio: A high-performance tool for negation and uncertainty detection in radiology reports. AMIA Jt. Summits Transl. Sci. Proc..

[39] Smit A., Jain S., Rajpurkar P., Pareek A., Ng A.Y., Lungren M.P. (2020). CheXbert: Combining automatic labelers and expert annotations for accurate radiology report labeling using BERT. arXiv.

[40] Chapman W.W., Bridewell W., Hanbury P., Cooper G.F., Buchanan B.G. (2001). A simple algorithm for identifying negated findings and diseases in discharge summaries. J. Biomed. Inform..

[41] Xiao J., Li S., Lin T., Zhu J., Yuan X., Feng D.D., Sheng B. (2024). Multi-label chest X-ray image classification with single positive labels. IEEE Trans. Med. Imaging.

[42] Cole E., Mac Aodha O., Lorieul T., Perona P., Morris D., Jojic N. (2023). Multi-label learning from single positive labels. Proceedings of the IEEE/CVF Conference on Computer Vision and Pattern Recognition (CVPR).

[43] Jang J., Kyung D., Kim S.H., Lee H., Bae K., Choi E. (2024). Significantly improving zero-shot X-ray pathology classification via fine-tuning pre-trained image-text encoders. Sci. Rep..

[44] Tiu E., Talius E., Patel P., Langlotz C.P., Ng A.Y., Rajpurkar P. (2022). Expert-level detection of pathologies from unannotated chest X-ray images via self-supervised learning. Nat. Biomed. Eng..

[45] Kufel J., Bielówka M., Rojek M., Mitrȩga A., Lewandowski P., Cebula M., Krawczyk D., Bielówka M., Kondoł D., Bargieł-Łączek K. (2023). Multi-label classification of chest X-ray abnormalities using transfer learning techniques. J. Pers. Med..

[46] Huang G., Liu Z., van der Maaten L., Weinberger K.Q. (2017). Densely connected convolutional networks. Proceedings of the IEEE Conference on Computer Vision and Pattern Recognition (CVPR).

[47] Tan M., Le Q.V. (2019). EfficientNet: Rethinking model scaling for convolutional neural networks. Proceedings of the International Conference on Machine Learning (ICML).

[48] Ucan M., Kaya B., Aygun O., Kaya M., Alhajj R. (2025). Comparison of EfficientNet CNN models for multi-label chest X-ray disease diagnosis. PeerJ Comput. Sci..

[49] Xiong K., Tu Y., Rao X., Zou X., Du Y. (2025). Multi-label disease detection in chest X-ray imaging using a fine-tuned ConvNeXtV2 with a customized classifier. Informatics.

[50] Mosquera C., Ferrer L., Milone D.H., Luna D., Ferrante E. (2024). Class imbalance on medical image classification: Towards better evaluation practices for discrimination and calibration performance. Eur. Radiol..

[51] Hanif M.S., Bilal M., Alsaggaf A.H., Al-Saggaf U.M. (2025). Enhancing multi-label chest X-ray classification using an improved ranking loss. Bioengineering.

[52] Yang S., Wu Y. (2025). Intelligent diagnosis model for chest X-ray images diseases based on convolutional neural network. BMC Med. Imaging.

[53] Zhang K., Liang W., Cao P., Mao Z., Yang J., Zaiane O.R. (2025). CorLabelNet: A comprehensive framework for multi-label chest X-ray image classification with correlation-guided discriminant feature learning and oversampling. Med. Biol. Eng. Comput..

[54] Guendel S., Grbic S., Georgescu B., Liu S., Maier A., Comaniciu D. (2019). Learning to recognize abnormalities in chest x-rays with location-aware dense networks. Proceedings of the Iberoamerican Congress on Pattern Recognition (CIARP).

[55] Öztürk Ş., Turalı M.Y., Çukur T. (2025). HydraViT: Adaptive multi-branch transformer for multi-label disease classification from chest X-ray images. Biomed. Signal Process. Control.

[56] Roy Reena M., Ameer P.M. (2024). Deep learning based classification of multi-label chest X-ray images via dual-weighted metric loss. Artif. Intell. Med..

[57] Ashraf S.N., Mamun M.A., Abdullah H.M., Alam M.G.R. (2023). SynthEnsemble: A fusion of CNN, Vision Transformer, and hybrid models for multi-label chest X-ray classification. Proceedings of the 26th International Conference on Computer and Information Technology (ICCIT), Cox’s Bazar, Bangladesh, 13–15 December 2023.

[58] Albahli S., Rauf H.T., Algosaibi A., Balas V.E. (2021). AI-driven deep CNN approach for multi-label pathology classification using chest X-Rays. PeerJ Comput. Sci..

[59] Bajwa N., Bajwa K., Shakeel M.F., Rana A., Haqqi K., Khan S. (2023). A generalized deep learning model for multi-disease chest X-ray diagnostics. Proceedings of the International Work-Conference on Artificial Neural Networks (IWANN), 2023.

[60] Thapa M., Kaur R., Mehta G., Wickramasinghe N., Kakkar D. (2024). Deep learning-based multi-label image classification for chest X-rays. Innovations in VLSI, Signal Processing and Computational Technologies.

[61] Ge Z., Mahapatra D., Chang X., Chen Z., Chi L., Lu H. (2020). Improving multi-label chest X-ray disease diagnosis by exploiting disease and health labels dependencies. Multimed. Tools Appl..

[62] Mohd Redzwan A., Yeoh P.S.Q., Lai K.W., Lai K.W., Yeoh P.S.Q. (2025). Multi-label convolutional neural network for multiple diseases detection in chest radiographs. Biomedical Engineering.

[63] Malik H., Anees T., Mui-zzud-din (2022). BDCNet: Multi-classification convolutional neural network model for classification of COVID-19, pneumonia, and lung cancer from chest radiographs. Multimed. Syst..

[64] Li L., Cao P., Yang J., Zaiane O.R. (2022). Modeling global and local label correlation with graph convolutional networks for multi-label chest X-ray image classification. Med. Biol. Eng. Comput..

[65] Wang H., Jia H., Lu L., Xia Y. (2019). Thorax-Net: An attention regularized deep neural network for classification of thoracic diseases on chest radiography. IEEE J. Biomed. Health Inform..

[66] Kabir S., Farrokhvar L., Dabouei A. (2023). A weakly supervised approach for thoracic diseases detection. Expert Syst. Appl..

[67] Chen B., Zhang Z., Li Y., Lu G., Zhang D. (2021). Multi-label chest X-ray image classification via semantic similarity graph embedding. IEEE Trans. Circuits Syst. Video Technol..

[68] Liu Z., Cheng Y., Tamura S. (2023). Multi-label local to global learning: A novel learning paradigm for chest X-ray abnormality classification. IEEE J. Biomed. Health Inform..

[69] Cai D., Lu H., Chai Z., Wang R., Zhu W., Yao Y. (2023). Label semantic improvement with graph convolutional networks for multilabel chest X-ray image classification. Proceedings of the 13th International Conference on Information Technology in Medicine and Education (ITME), 2023.

[70] Ibrahim D.M., Elshennawy N.M., Sarhan A.M. (2021). Deep-chest: Multi-classification deep learning model for diagnosing COVID-19, pneumonia, and lung cancer chest diseases. Comput. Biol. Med..

[71] Mahmud T., Rahman M.A., Fattah S.A. (2020). CovXNet: A multi-dilation convolutional neural network for automatic COVID-19 and other pneumonia detection from chest X-ray images with transferable multi-receptive feature optimization. Comput. Biol. Med..

[72] Yang M., Tanaka H., Ishida T. (2023). Performance improvement in multi-label thoracic abnormality classification of chest X-rays with noisy labels. Int. J. Comput. Assist. Radiol. Surg..

[73] Chen Y., Wan Y., Pan F. (2023). Enhancing multi-disease diagnosis of chest X-rays with advanced deep-learning networks in real-world data. J. Imaging Inform. Med..

[74] Ouyang X., Karanam S., Wu Z., Chen T., Huo J., Zhou X.S., Wang Q., Cheng J.Z. (2020). Learning hierarchical attention for weakly-supervised chest X-ray abnormality localization and diagnosis. IEEE Trans. Med. Imaging.

[75] Mao C., Yao L., Luo Y. (2022). ImageGCN: Multi-relational image graph convolutional networks for disease identification with chest X-rays. IEEE Trans. Med. Imaging.

[76] Minaee S., Kafieh R., Sonka M., Yazdani S., Soufi G.J. (2020). Deep-COVID: Predicting COVID-19 from chest X-ray images using deep transfer learning. Med. Image Anal..

[77] Dosovitskiy A., Beyer L., Kolesnikov A., Weissenborn D., Zhai X., Unterthiner T., Dehghani M., Minderer M., Heigold G., Gelly S. (2021). An image is worth 16x16 words: Transformers for image recognition at scale. Proceedings of the International Conference on Learning Representations (ICLR).

[78] Touvron H., Cord M., Douze M., Massa F., Sablayrolles A., Jégou H. (2021). Training data-efficient image transformers & distillation through attention. Proceedings of the International Conference on Machine Learning (ICML).

[79] Liu Z., Lin Y., Cao Y., Hu H., Wei Y., Zhang Z., Lin S., Guo B. (2021). Swin transformer: Hierarchical vision transformer using shifted windows. Proceedings of the IEEE International Conference on Computer Vision (ICCV).

[80] Cao H., Wang Y., Chen J., Jiang D., Zhang X., Tian Q., Wang M. (2022). Swin-UNet: UNet-like pure transformer for medical image segmentation. Proceedings of the European Conference on Computer Vision (ECCV) Workshops.

[81] Gu A., Zhang H., Huang M., Xu Z., Yu L., Liu J., Wang Y. (2022). SwinCheX: Multi-label classification on chest X-ray images with transformers. arXiv.

[82] Chen J., Lu Y., Yu Q., Luo X., Adeli E., Wang Y., Lu L., Yuille A.L., Zhou Y. (2021). TransUNet: Transformers make strong encoders for medical image segmentation. arXiv.

[83] Steiner A., Kolesnikov A., Zhai X., Wightman R., Uszkoreit J., Beyer L. (2021). How to train your ViT? Data, augmentation, and regularization in vision transformers. arXiv.

[84] Park S., Kim G., Oh Y., Seo J.B., Lee S.M., Kim J.H., Moon S., Lim J.-K., Ye J.C. (2022). Vision transformer for classification of breast ultrasound images and chest X-rays. Proceedings of the IEEE International Symposium on Biomedical Imaging (ISBI).

[85] Li X., Liu Y., Xu X., Zhao X. (2025). CheX-DS: Improving chest X-ray image classification with ensemble learning based on DenseNet and Swin Transformer. arXiv.

[86] Kim D. (2023). CheXFusion: Effective fusion of multi-view features using transformers for long-tailed chest X-ray classification. Proceedings of the IEEE/CVF International Conference on Computer Vision Workshops (ICCVW).

[87] Naseer M., Ranasinghe K., Khan S., Hayat M., Khan F.S., Yang M.-H. (2021). Intriguing properties of vision transformers. NIPS’21: Proceedings of the 35th International Conference on Neural Information Processing Systems.

[88] Tuli S., Dasgupta I., Grant E., Griffiths T.L. (2021). Are convolutional neural networks or transformers more like human vision?. arXiv.

[89] Kim H., Kim T., Kim S., Lee M., Hwang D. (2023). CheSS: Chest X-Ray Pre-trained Model via Self-supervised Contrastive Learning. J. Imaging Inform. Med..

[90] Shakouri M., Iranmanesh F., Eftekhari M. (2023). DINO-CXR: A Self Supervised Method Based on Vision Transformer for Chest X-Ray Classification. Advances in Visual Computing (ISVC 2023).

[91] Huang W., Li C., Zhou H.-Y., Yang H., Liu J., Liang Y., Zheng H., Zhang S., Wang S. (2024). Enhancing representation in radiography-reports foundation model: A granular alignment algorithm using masked contrastive learning. Nat. Commun..

[92] Huijben E.M.C., Pluim J.P.W., van Eijnatten M.A.J.M. (2024). Denoising diffusion probabilistic models for addressing data limitations in chest X-ray classification. Inform. Med. Unlocked.

[93] Baek S., Shin J. (2025). Counterfactual Augmentation for Long-Tailed Multi-label Chest X-Ray Classification. Efficient Medical Artificial Intelligence (EMAI).

[94] Zhou W., Tang B., Cong R., Jiang Q. (2026). Turbidity–Similarity Decoupling: Feature-Consistent Mutual Learning for Underwater Salient Object Detection. IEEE Trans. Image Process..

[95] Zhou W., Li Y., Jiang Q., Liao L., Cong R., Lin W. (2026). Decouple-Then-Synergize: A Self-Paced Collaborative Learning Network for RGB-T Snowy Urban Scene Parsing. IEEE Trans. Image Process..

[96] Zhou W., Ju Z., Cong R., Yan W. (2026). RCNet: Dual-Network Resonance Collaboration via Mutual Learning for RGB-D Road Defect Detection. IEEE Trans. Circuits Syst. Video Technol..

[97] Zhou W., YWu u., Qiang F., Yan W. (2026). Lightweight Scope Integration Network for Rail Surface Defect Detection. IEEE Trans. Big Data.

[98] Buda M., Maki A., Mazurowski M.A. (2018). A systematic study of the class imbalance problem in convolutional neural networks. Neural Netw..

[99] Yagis E., Atnafu S.W., García Seco de Herrera A., Marzi C., Scheda R., Giannelli M., Tessa C., Citi L., Diciotti S. (2021). Effect of data leakage in brain MRI classification using 2D convolutional neural networks. Sci. Rep..

[100] Sundaram S., Hulkund N. (2021). GAN-based data augmentation for chest X-ray classification. Proceedings of KDD DSHealth.

[101] Kowald D., Scher S., Pammer-Schindler V., Müllner P., Waxnegger K., Demelius L., Fessl A., Toller M., Mendoza Estrada I.G., Šimić I. (2024). Establishing and evaluating trustworthy AI: Overview and research challenges. Front. Big Data.

[102] Nastoska A., Jancheska B., Rizinski M., Trajanov D. (2025). Evaluating trustworthiness in AI: Risks, metrics, and applications across industries. Electronics.

